# Hydrogels for localized chemotherapy of liver cancer: a possible strategy for improved and safe liver cancer treatment

**DOI:** 10.1080/10717544.2022.2070299

**Published:** 2022-05-09

**Authors:** Jianyong Ma, Bingzhu Wang, Haibin Shao, Songou Zhang, Xiaozhen Chen, Feize Li, Wenqing Liang

**Affiliations:** a Department of General Practice, Shaoxing People’s Hospital, Shaoxing Hospital of Zhejiang University, Shaoxing, China; b Internal Medicine of Integrated Traditional Chinese and Western Medicine, Zhoushan Hospital of Traditional Chinese Medicine Affiliated to Zhejiang Chinese Medical University, Zhoushan, China; c College of Medicine, Shaoxing University, Shaoxing, China; d Medical Research Center, Zhoushan Hospital of Traditional Chinese Medicine Affiliated to Zhejiang Chinese Medical University, Zhoushan, China

**Keywords:** Hydrogel, stimuli-responsive, liver cancer, chemotherapy, ligands, polymer

## Abstract

The systemic drug has historically been preferred for the treatment of the majority of pathological conditions, particularly liver cancer. Indeed, this mode of treatment is associated with adverse reactions, toxicity, off-target accumulation, and rapid hepatic and renal clearance. Numerous efforts have been made to design systemic therapeutic carriers to improve retention while decreasing side effects and clearance. Following systemic medication, local administration of therapeutic agents allows for higher ‘effective’ doses with fewer side effects, kidney accumulation, and clearance. Hydrogels are highly biocompatible and can be used for both imaging and therapy. Hydrogel-based drug delivery approach has fewer side effects than traditional chemotherapy and can deliver drugs to tumors for a longer time. The chemical and physical flexibility of hydrogels can be used to achieve disease-induced *in situ* accumulation as well as subsequent drug release and hydrogel-programmed degradation. Moreover, they can act as a biocompatible depot for localized chemotherapy when stimuli-responsive carriers are administrated. Herein, we summarize the design strategies of various hydrogels used for localized chemotherapy of liver cancer and their delivery routes, as well as recent research on smart hydrogels.

## 1. Introduction

Liver cancer, the world's leading cause of cancer death and the fifth leading cause in the USA is the only one of the top five most lethal cancers to see an annual percentage increase in occurrence (Miller et al., 2019). Hepatocellular carcinoma (HCC) contributes to 80–90% of all primary liver cancers in adults, while cholangiocarcinoma (CCA) accounts for 10–15% (Ringelhan et al., 2018). Children and adolescents are also affected by liver cancer, with hepatoblastoma (HB) and HCC accounting for 67–80% and 20–33% of cases, respectively (Sharma, 2020). Globally, the number of newly diagnosed cases and the age-standardized incidence rate of liver cancer have increased over the last few decades, despite substantial public health efforts to combat this problem. Liver diseases are more prevalent in developing countries (Starley et al., 2010) and risk factors include fatty liver disease, hepatitis B and C viruses, alcohol-related cirrhosis, obesity, smoking, diabetes, iron overload, and numerous dietary exposures (Center & Jemal, 2011). Despite this, various *de novo* cases in non-cirrhotic patients have invalidated the theory that it only occurs in cirrhotic livers. HCC has also been reported in patients who did not have any signs of cirrhosis and had no histological evidence of hepatitis (Mittal et al., 2016; Pennisi et al., 2019). Liver cancer has a poor prognosis. Only small proportion of patients (almost 5–15%) are candidates for surgical removal and appropriate for early-stage patients only with impaired hepatic regenerative capacity, usually without cirrhosis; right hepatectomy has a higher risk of post-operative complications than left hepatectomy. The following are some treatments available for more advanced stages: (a) trans-arterial chemoembolization (TACE), which improves 2-year survival for intermediate-stage HCC patients by 23% when compared to conservative therapy. (b) Sorafenib oral dosing, a kinase inhibitor that is the most commonly prescribed treatment for late-stage cancers. However, the treatment benefits only about a third of patients, and drug resistance develops in six months of starting the regimen (El-Serag et al., 2008). Chemotherapeutic drugs, like sorafenib, can cause toxicity and/or drug inefficacy if used over an extended period of time. Therefore, current ablation and chemotherapy treatments are ineffective toward improved prognosis of this deadly disease. More research is needed to discover improved techniques to treat liver cancer.

**Figure 1.**
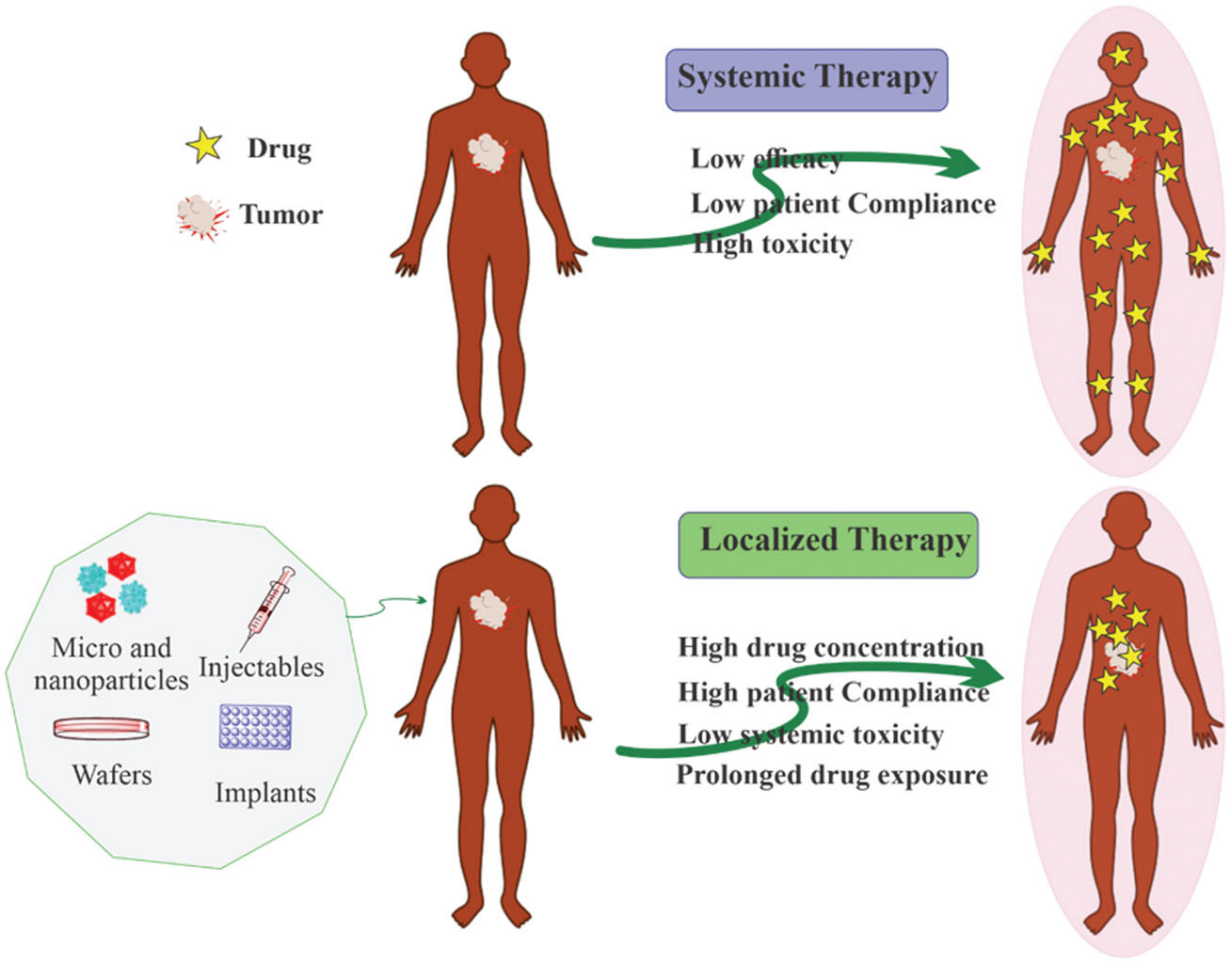
Advantages of localized chemotherapy over conventional systemic chemotherapy for the treatment of solid tumors.

Chemotherapy, which employs highly toxic chemotherapeutic chemicals against cancer cells, is the preferred therapeutic method for various cancer managements because of its superior efficacy (Qin et al., 2017). Although there are significant limitations to systemic chemotherapy, it is the most often employed therapeutic approach. It entails administering anti-cancer medications intravenously at maximal tolerable levels, which causes significant toxicity to healthy cells ranging from neutropenia to cardiomyopathy (Scharovsky et al., 2009). Furthermore, systemic chemotherapy is inefficient at delivering drugs to therapeutic concentrations at target sites, and maintaining appropriate drug concentrations within tumors is difficult (Jang et al., 2003). Hence, only a small fraction of the injected amount reaches the tumor site, resulting in decreased therapeutic potential and increased damage to healthy cells. Hence, the use of localized delivery in cancer therapy has increased. Localized delivery can be defined as a way of delivering a drug from a dosage form to a specific location inside the biological system where the maximum pharmacological impact is desired. Ye et al. incorporated polylactic acid microspheres carrying epirubicin into collagen film intra-tumoral administration. The mouse liver cancer model showed significantly increased tumor suppression upon intra-tumoral implantation of microspheres compared with epirubicin delivered intra-tumorally alone (De Souza et al., 2010). This review is focused on localized chemotherapy either via intratumoral injection or the delivery of therapeutics in close proximity to the tumor site(s). Drug accumulation at target sites significantly reduces their systemic exposure and is considered a key factor for minimum toxicity and maximum efficacy. In this regard, biomaterials would be beneficial for keeping the drug at the targeted sites (Figure 1).

Hydrogels are polymer networks with a hydrophilic character that are three-dimensionally crosslinked. They can easily swell in water and retain a huge amount of water while swollen. They can change volume dramatically under the influence of a wide range of physical, chemical, and biological stimuli. Temperature, electric field, magnetic field, solvent composition, pressure, light, and sound are all examples of physical stimuli, whereas pH, ions, and specific molecular recognition events are all examples of chemical or biological stimuli (Qiu & Park, 2001; Miyata et al., 2002). In this regard, hydrogel would be considered safe with improved efficacy in cancer treatment of the liver. Hydrogels have become a hot topic in recent years, with about 5000 papers published in 2010, which is nearly five times the amount of papers published in 2010 linked to ‘nanotechnology’, also lately become a most focused area in the scientific community (Lee et al., 2013). Hydrogels prevalence is due to four characteristics: biocompatibility, biodegradability, loading capacity of drug entity, and controlled release profile. Biocompatibility suggests that the hydrogels are not associated with significant cellular or systemic toxicity in the body upon its implantation, and are not included in stimulating the immune system. Moreover, many of the polymers used to make hydrogels for cancer therapy are biodegradable, including natural polymers and synthetic polymers (e.g. chitosan (CTS) (Jamal et al., 2018), hyaluronic acid (HA) (Cho et al., 2015), alginate (ALG) (Shih et al., 2018), polyesters (Shen et al., 2017), and polyphosphazene (PPZ) (Society, 2018)) which are rapidly depleted by cells and remodeled in the body after administration (Yamaguchi et al., 2007). US Patent 5,514,380 describes the use of nontoxic and biodegradable hydrogel based on multi-block copolymers as drug delivery vehicles. They were made from a biodegradable, hydrophobic hard block and a hydrophilic soft block. The hydrolysis of intramolecular amide and ester bonds, which occurs naturally in the human body, could be used to degrade them. Copolymers PEO and polypropyleneoxide (PPO) and/or polyethyleneoxide (PEO) with 600–30,000 Da of molecular weight (MW) have essential properties for the hydrophilic, non-biodegradable polymers used in the aforementioned patent. In this regard, polyglycolide (PGA), polylactide (PLA), or a PLA/PGA copolymer are being used as biodegradable polymers (Song et al., 1996). Additionally, hydrogels, due to their porous nature, can carry huge content of water-soluble substances, i.e. peptides and proteins (Vermonden et al., 2012). Additionally, the release rate of drugs can be regulated by altering the structure of hydrogels (Sepantafar et al., 2017).

Hydrogels are large polymeric networks that are highly magnified, hydrophilic, and capable of retaining a large amount of water within their pores (Martin & Youssef, 2018; Wang, 2018). Due to their enhanced biocompatibility, these bioactive resources are commonly used in tissue engineering (Vermonden & Klumperman, 2015). Hydrogels have a porous structure and soft surface and behave similarly to natural living tissues. Owing to various stimuli-responsive potential to temperature, pH, pressure, electric and magnetic fields, these hydrogels are undoubtedly referred to as smart materials. Even at a slight pH change in the swelling medium, these materials have a high potential for proton release and uptake (Liu et al., 2015). The above mention characteristic of hydrogels makes them able to be served as an effective drug delivery vehicle. Literature has demonstrated the remarkable contributions of multi-responsive hydrogels such as polyvinyl alcohol crosslinked polyacrylamide and poly(N-isopropylacrylamide) (PNIPAM)/CTS as smart sensors, effectors, and targeted delivery to tumor cells (Liu et al., 2017; Manga & Jha, 2017). Polymeric hydrogels such as CTS and keratin (polylactic keratin) are extensively studied for their potential use in the fabrication of LCD (liquid crystal display) and OLED (organic light-emitting diode) materials, as well as specific drug delivery systems (Karimi et al., 2017).

Although hydrogels vary greatly in shape and size, drug release behavior are similar due to analogous interactions between polymer networks and drugs. When compared to normal tissue, various distinct features found in tumor tissues (e.g. hypoxia, enhanced retention, and permeability effects as well as acidic pH) can be utilized to design drug carriers (Danhier et al., 2010). In general, cancer treatment hydrogels can be designed in a variety of sizes, ranging from nanogels to macroscopic gels (Yallapu et al., 2011), as well as injectable hydrogels (*in situ*) (Norouzi et al., 2016), and can be developed to demonstrate multiple drug mechanisms in external and internal stimuli. In this paper, various types of hydrogels and their administration routes for liver cancer treatment will be discussed and we review various drug release behavior and hydrogel formation and measure the efficacy of hydrogels as a controlled drug carrier and their future prospects.

## 2. Advantage of hydrogels in drug delivery

Various definitions have been proposed for hydrogels over the years and are frequently described as a cross-linked polymeric network formed via conjugation or reaction of one or more monomers and possess water-swollen characteristics. Though, hydrogels are three-dimensional networks having potential for water absorption because of the existence of hydrophilic functionalities capable to fill the space between macromolecules and possess enhanced affinity toward biological fluids (Chai et al., 2017). Hydrogel-based advanced dressings have been shown to be ominously effective toward wound healing because of their moisture maintaining potential at the application site, preventing fluids from spreading to other healthy skin areas. Various hydrogels are now available commercially, including DermaFilm^®^, Condress^®^, Kaltostat^®^, and Sofargen^®^ (Tavakoli & Klar, 2020), with some containing ingredients such as zinc or iodine ions, which have cleansing and antimicrobial properties, respectively (Caló & Khutoryanskiy, 2015).

Drug delivery is one application where hydrogels special physical properties have gained attention. Controlling cross-link density in the gel matrix and affinity of hydrogels for the aqueous environment can be used to fine-tune their highly porous structure (Schiller & Lai, 2020). This allows drugs to be loaded into their gel matrix and then released in an amount that is dependent on the diffusion coefficient of small molecules or macromolecules. Due to their pharmacokinetic properties, it is possible to use hydrogels for drug delivery that is primarily focused on maintaining a high local concentration of the drug in the surrounding tissues for an extended period, while they can also be used for systemic delivery. Due to their biocompatibility, hydrogels can be used in the peritoneum and other areas of the body (Sultana et al., 2019). Hydrogels high-water content, as well as their physicochemical similarity to the natural extracellular matrix and mechanical properties, improves biocompatibility. It is possible to develop hydrogels that are biodegradable or dissolvable via environmental, hydrolytic, or enzymatic pathways, although this may not be desirable regarding the time and placement of the drug delivery device. Hydrogels, on the other hand, are somewhat pliable and may be molded to fit any surface. The muco- or bio-adhesive characteristics of some hydrogels can be helpful in the application of these hydrogels on non-horizontal surfaces or in the immobilization of these hydrogels at the application site. Rosiak et al. utilized gamma radiation to cross-link natural polymers (e.g. agar or gelatin) and synthetic polymers (e.g. poly(vinyl pyrrolidone) (PVP) or poly(vinyl alcohol) (PVA)) to produce sterile hydrogels for wound treatment. Their hydrogels are currently manufactured and marketed under the brand names 'Kikgel' and 'Aqua-gel' wound dressings (Rosiak et al., 1989). Hydrogels are being patented for contact lenses, in addition to the wound dressing. When macromonomers are used to make hydrogels, the necessity for their purification may be eliminated, because the used materials are generally nontoxic. For the synthesis, pH-sensitive polymers were used (hydroxyethyl)methacrylate-co-methacrylic acid, allowing for the production of hydrogels capable of undergoing a volume phase transition at a particular pH. This can be done by using salt solutions like sodium phosphate or sodium bicarbonate in the device in order to change the microenvironment and initiate the release of active ingredient. This solution's pH might be anywhere from 7.5–8.4 or 6.4–7.3, which could cause the hydrogel to either expand or contract (Rosenthal et al., 2006).

As hydrogels have been successfully used in various biomedical applications, it was hypothesized that they have the potential to be drug delivery platforms in cancer therapy. In cases of nonsquamous or incompletely removed tumors, local drug administration may be extremely beneficial even though most cancer treatment research focuses on systemic and oral administration. In this frame of reference, the *in situ* application of hydrogels, particularly stimuli-responsive hydrogels, at the tumor site/cavity has already been postulated, to promote local sustained drug release while reducing off-target effects and systemic exposure (Puente et al., 2018). Furthermore, using depot systems reduces the need for multiple cycles for cancer therapy, which has several negative consequences for patients as well as a financial impact on healthcare systems (Wei et al., 2020). Hydrogels that respond to various stimuli like temperature, light, pH, magnetic fields, and ionic strength are promising smart materials (Qu et al., 2018). US Patent 8,383,153 B2 demonstrates a drug carrier based on poly(amidoamine) based hydrogel. This temperature- and pH-sensitive hydrogel possessed a structure designed for prolonged drug release rather than burst release. The substance can be synthesized in a single step by connecting the secondary amine groups (NH) of a diamine molecule (such as piperazine) and vinyl groups (CH_2_CH) of an alkylene bisacrylamide compound. This hydrogel can be employed as a vehicle for a variety of physiologically active compounds via diverse administration routes (Lee et al., 2013). Such kind of hydrogel has received prominence due to the possibility of manipulating the hydrogel's rheological behavior in response to the various conditions of the tumor microenvironment. Indeed, cancer cells exhibit a variety of metabolic adaptive responses to ensure their survival. Nutrient and glucose uptake, lactic acid production under aerobic circumstances, and adaptation to hypoxic and microenvironments containing less amount of nutrient are just a few of them. In addition, cancer cells have a low pH extracellular environment and a high pH intracellular cytoplasm (Persi et al., 2018). As a result of these characteristics, it is possible for the design of delivery carrier with stimuli-responsive behavior for tumor-targeting (Table 1).

**Table 1.** Hydrogel-based drug delivery systems for cancer treatment.

Delivery route	Drug	Hydrogel	Cell model (*in vitro*)	Cancer model (*in vivo*)	Feature	Ref.
*In situ* injection	Temozolomide and paclitaxel	PEG-DMA	U87MG	Glioblastoma	Codelivery of temozolomide and paclitaxel	Zhao et al. (2019)
	PTX	GC-β-CD	SKOV3	Ovarian cancer	Conjugated β-CD for photocuring	Hyun et al. (2019)
Intratumoral injection	DOX and MBP	Chitosan	HT29	Colon cancer	Chemotherapy and hyperthermia combined	Zheng et al. (2019)
	Paclitaxel	FER-8 peptide	HepG2	Liver cancer	High drug-loading, sustained release, and higher degradation	Raza et al. (2019)
Intravenous administration	DOX	Dextrin	4T1 and U2OS	Breast cancer	Effectively targeting and inhibiting cancer progression	Zhang et al. (2017)
	Doxorubicin	PAA	SMCC-7721	Liver cancer	Stabilized amorphous CaCO_3_ and tunable pH-responsiveness	Xu et al. (2019)
	DOX and ICG	NIPAM, SBMA, MAA, and BAC	HepG2	Liver cancer	Near-infrared light-triggered and temperature/redox-sensitive	Li et al. (2017)
Subcutaneous injection	DOX/IL-2/IFN-γ	PELG-PEG-PELG	B16F10	Melanoma	Localized codelivery of anticancer drugs	Lv et al. (2018)
	AD-DOX	Poly(NIPAM-*co*-MPCD)	MCF-7 and HeLa	Sarcoma	pH/NIR dual-sensitive for chemo-photothermal treatment	Xu et al. (2017)
Transdermal delivery	Silibinin	PCBCL-*b*-PEG-*b*-PCBCL	B16F10	Melanoma	Patch loaded with silibinin for the treatment of melanoma	Makhmalzadeh et al. (2018)
Peritumoral injection	Cisplatin and CA4P	mPEG-*b*-PELG	CT26 and MCF-7	Colorectal cancer	CDDP and CA4P coloaded polypeptide gel	Yu et al. (2019)
Intraperitoneal injection	Oxaliplatin and tannic acid	PCL-10R5-PCL	CT26	Colorectal cancer	Oxaliplatin coloaded with tannic acid	Ren et al. (2019)

PEG-DMA: polyethylene glycol dimethacrylate; GC: glycol CTS; CD: cyclodextrin; PF127: Pluronic F127; DOX: doxorubicin; PAA: poly(acrylic acid); ICG: indocyanine green; NIPAM: n-isopropylacrylamide; SBMA: sulfobetaine methacrylate; MAA: methylallyl amine; BAC: N,N′-bis(acryloyl)cystamine; PELG: poly(γ-ethyl-l-glutamate); PCBCL: poly[(α-benzyl carboxylate-ε-caprolactone)-co-(α-carboxyl-ε-caprolactone)]; PCL: poly(3-caprolactone).

Numerous hydrogel-based formulations have been developed over the years for cancer prevention, diagnosis, and treatment, with several of them being enrolled in clinical trials. Additionally, several have received European Medicines Agency (EMA) and/or FDA approval and have been promoted to clinical practice such as Lupron^®^ depot or Eligard^®^, two hydrogels based on PLGA for *in situ* delivery of leuprolide acetate to treat prostate cancer of advanced stage (Wei et al., 2020). TraceIT^®^ is another example. It is a microparticle of PEG hydrogel covalently bound with iodine that has been approved as a tissue marker. Once injected, it enables precise tumor visualization via computed tomography (CT), magnetic resonance imaging (MRI), and ultrasound, allowing for a more precise cancer cells identification prior to surgery. Additionally, it is also helpful to identify more precise dose selection in radiation therapy, thereby avoiding normal sites. The product maintains its dimensional stability for three months before being completely absorbed after six months (Bair et al., 2015). TraceIT^®^'s feasibility and efficacy as an organ spacer are being evaluated in clinical trials for vaginal or prostate sparing, rectal cancer treatment, and also as a spacer in pancreatic cancer patients between their pancreas and duodenum (Andrade et al., 2021). SpaceOAR^®^, a PEG-based hydrogel is another example of an organ spacer approved by the FDA for use in preventing rectum injury during the session of radiation therapy in patients with prostate cancer (Velde et al., 2019).

## 3. Classification and preparation of hydrogels

### 3.1. Classification of hydrogels

Classification of hydrogel has been reported on the various basis including the following.

#### 3.1.1. Classification according to the source

Two different categories of hydrogels are present according to their natural or synthetic origins (Zhao et al., 2013).

#### 3.1.2. Classification based on the composition of the polymer

Some major hydrogel classes are formed as a result of the preparation method. Following are the example of these:

- *Homopolymeric hydrogels*: Monomers are the fundamental constituents of polymer networks, and homopolymeric hydrogels are networks composed entirely of a single monomer species (Iizawa et al., 2007). Cross-linked structures can be found in homopolymers depending on the nature of their monomer and the method used for polymerization.
- *Copolymeric hydrogels*: Copolymeric hydrogels are composed of two or more distinct monomer species that contain at least one hydrophilic component. The polymer network may have a random, block, or alternating configuration (Yang et al., 2002).
- Multipolymer interpenetrating polymeric hydrogel (IPN), a type of hydrogel, is composed of two independent cross-linked synthetic and/or natural polymer components held together in a network form. One component of semi-IPN hydrogel is a cross-linked polymer, while the other is a non-cross-linked polymer (Maolin et al., 2000).

#### 3.1.3. Configuration-based classification

Hydrogels can be classified depending on their structure and composition as follows: amorphous (non-crystalline), crystalline, and semicrystalline (a complex blend of crystalline and amorphous phases).

#### 3.1.4. Classification according to the nature of the cross-linking

Hydrogels are classified into two types based on the chemical or physical cross-linked junctions. Permanent junctions exist in chemically cross-linked networks, whereas transient junctions occur in physical networks as a result of entanglements of the polymer chain or physical interactions such as hydrogen bonds, ionic interactions, or hydrophobic interactions (Hacker et al., 2019).

#### 3.1.5. Classification according to network electrical charge

**Figure 2.**
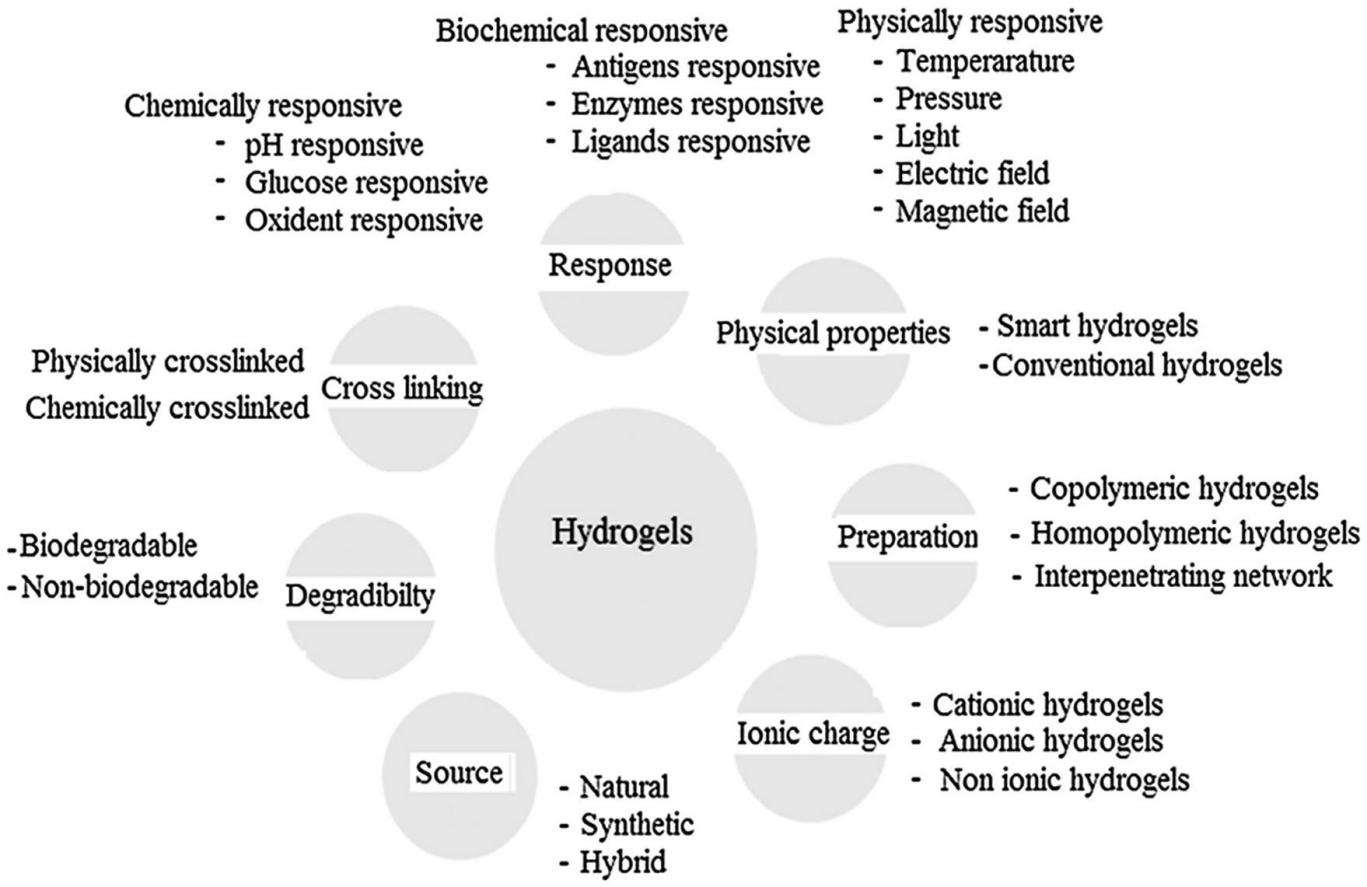
Classification of hydrogels based on the different properties. Reproduced with permission from reference Ullah et al. (2015).

There are four types of hydrogels based electrical charges on the cross-linked chains including nonionic, ionic amphoteric electrolyte, and zwitterionic hydrogels (Figure 2).

### 3.2. Synthesis of hydrogel

From a wide range of monomeric and polymeric constituents, hydrogels are produced such as acrylamide (Zhang et al., 2016) polysaccharides, viz. CTS, starch, cellulose (Abdel-Halim & Al-Deyab, 2014; An et al., 2015; Godiya et al., 2019), ALG (Sahraei & Ghaemy, 2017), gums (Ding et al., 2018), cellulose and rice husk (Gharekhani et al., 2017). Generally, the preparation of hydrogels utilizes either synthetic or natural polymers. When compared to conventional hydrogels, crosslinked junction hydrogels have unique and improved properties such as electrical, thermal, variable MW specific functionality with desired chemical content, and mechanical properties. Alginate-methylcellulose hydrogels that have been blended and cross-linked have shown to be promising materials for encapsulating and delivering olfactory unsheathing cells (OECs). They can be successfully used as a promising system for cell delivery owing to their highest encapsulation potential (Surrao et al., 2020). Electrostatic interactions between two naturally occurring polysaccharides, CTS, and HA resulted in polyelectrolyte multilayers and macroscopic hydrogels. Self-healing properties have been reported in such polyelectrolyte complexes, which can be used in biomedical applications (Barroso et al., 2019). Interpolymer complexes are formed as a result of the association between these poly-ions, which are regulated by specific bonding systems such as electrostatic interaction and hydrogen bonding between distinct polymer chains. Hydrogels are divided into two groups based on the types of cross-link junctions: chemically cross-linked and physically cross-linked.

#### 3.2.1. Synthesis of physically cross-linked hydrogel

In physically cross-linked hydrogels, ionic/electrostatic interactions, hydrogen bonding, amphiphilic polymers self-assembly, crystallization, interactions of protein, and metal coordination are the key physical interactions between polymer chains (Hu et al., 2019). For example, polyacrylic and polymethacrylic acids form hydrogen-bonded complexes with PEG. They are formed only when carboxylic acid groups undergo protonation, resulting in pH-sensitive hydrogel formation (Siqueira et al., 2018). Alginate is also a natural polymer that forms a gel when it comes into contact with divalent cations, for example, Ca^2+^ or Mg^2+^ (Hu et al., 2019). Thermally induced phase transitions in amphiphilic polymers, such as poloxamers, result in hydrogel formation owing to hydrophobic interactions occurring above the lower critical solution temperature (LCST) (Russo & Villa, 2019). Hydrogels based on PVA are an example of physical crosslinking induced by crystallization, as polymer undergoes crystallization and gelation as a result of repeated freeze–thaw of a solution (Kumar & Han, 2017). The main disadvantage of hydrogels obtained via physical crosslinking is their potential lack of or compromised stability under physiological environments. Therefore *in vivo* application, chemical crosslinking is preferred (Siqueira et al., 2018).

#### 3.2.2. Synthesis of chemically cross-linked hydrogel

**Figure 3.**
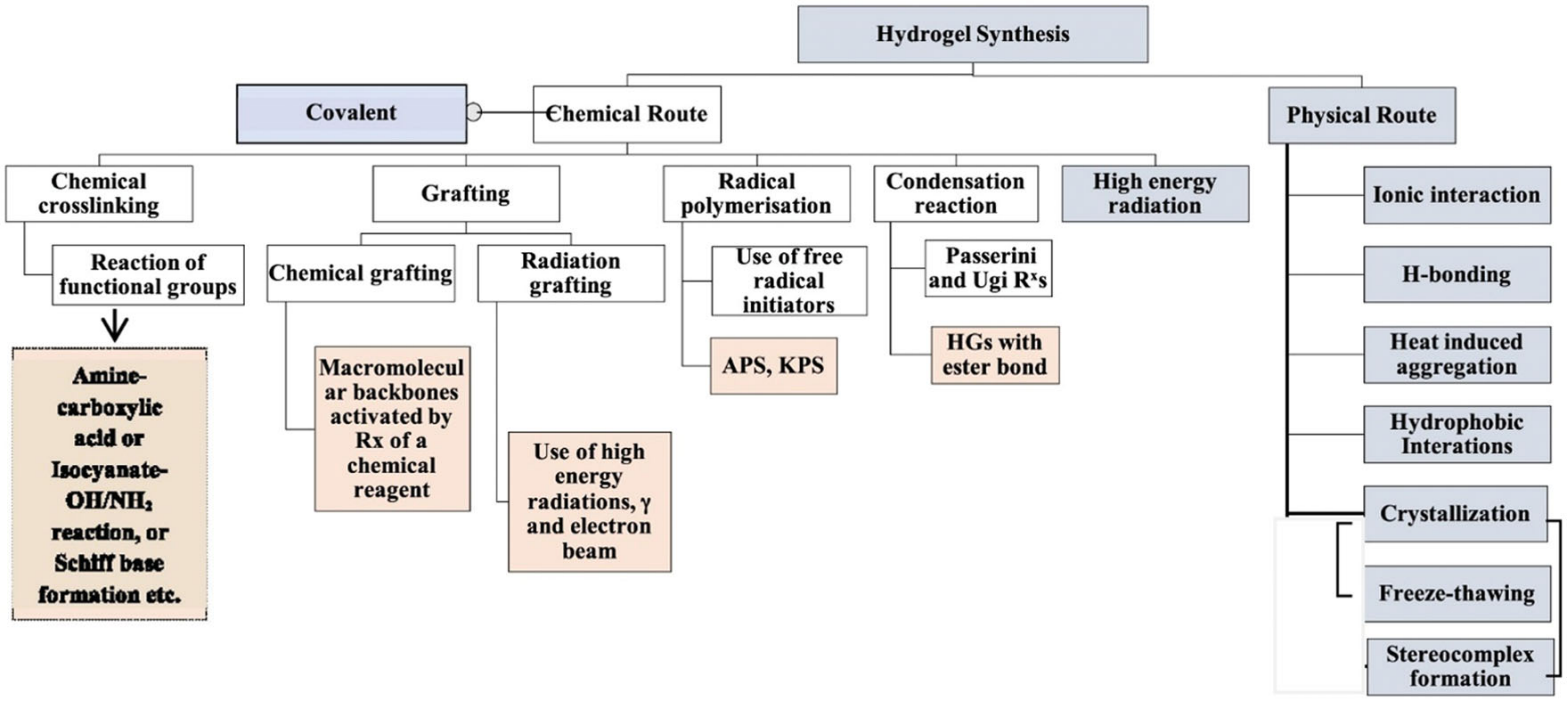
Various physical and chemical approaches for the synthesis of the hydrogel. Reproduced with permission from reference Sinha & Chakma (2019).

Due to the covalent linkages between the polymeric chains, chemically crosslinked hydrogels exhibited remarkable mechanical properties and were found to be more durable under physiological. Additionally, their degradation behavior is controllable, even though it is typically irreversible. In comparison to physically cross-linked hydrogels, a potential disadvantage is the frequent requirement of catalysts and organic solvents, which commonly generate concerns regarding biocompatibility and environmental influence (Hu et al., 2019). Click chemistry, which includes the formation of Schiff base, photopolymerization, Michael addition or Diels–Alder reaction, reversible addition-fragmentation chain transfer (RAFT) polymerization, free radical polymerization (FRP), or enzyme-catalyzed reactions, is commonly used to promote covalent bonds in chemically cross-linked hydrogels (Hu et al., 2019). Owing to the existence of certain groups in the hydrophilic polymers such as COOH–, NH2–, and OH–, isocyanate-OH/NH_2_ reaction, the formation of amine-carboxylic acid, or Schiff base is frequently used to covalently link polymers together to form the network of the hydrogel. Schiff base is a chemical compound that is frequently used in the formation of hydrogels and is typically formed when amines react nucleophilically with the electrophilic carbon of ketones or aldehydes. For instance, glycol CTS has been reported to be cross-linked with PEG, which led to the formation of promising hydrogels to deliver anticancer drugs (Hu et al., 2019). Due to the existence of unsaturated groups, acrylate derivatives (e.g. glycidylmethacrylate (GMA) and PEG diacrylate (PEGDA)) are frequently used as pre-polymers in photo-polymerization cross-linking. Photo-initiators (visible or UV light) such as Irgacure 2959, or Eosin-Y, among others, react with these groups. Under the influence of light, photons cleave photoinitiators, generating free-radical which in turn react with the vinyl bonds of pre-polymer, thereby facilitating crosslinking between the polymer chains (Sultana et al., 2019). For example, a transglutaminase enzyme catalyzes the reaction between the -carboxamide group (glutamine modified PEG) and the ‘-amine’ group resulting in the formation of an amide bond, which can be used to make PEG-based hydrogels (Choi et al., 2019) (Figure 3).

## 4. Hydrogels for localized chemotherapy of liver cancer

Different techniques and approaches are used for liver cancer patients depending on their diagnosis and stage which include chemotherapy, surgery, radiation and immunotherapy, gene therapy and targeted therapy (Bykov et al., 2018; Senft et al., 2018). Among these approaches, chemotherapy is critical for tumor control and recurrence prevention. Chemotherapeutic medicines' anti-tumor action kills tumor cells by causing drug toxicity (Zhou et al., 2017). Additionally, chemotherapeutic drugs that were directly injected into a tumor undergo frequent removal from the blood circulation, implying that the drugs could not be accumulated in tumor tissue for an extended time, defeating the goal of tumor elimination (Wang et al., 2015). Chemical medications may harm normal tissues as well as malignant cells if they are not selective or targeted. Traditional chemotherapeutic medications are acquiring new life due to the invention of the hydrogel. Therefore, several chemotherapeutic agents are encapsulated in hydrogel and injected into the tumor site to prolong the drug's content in the tumor tissue, minimize systemic toxicity, and prevent the discomfort of multiple injections (Takimoto & Awada, 2008). Hydrogels are an efficient drug delivery strategy because of the presence of an excess of water and the network of cross-linked polymer that has excellent biocompatibility, low cytotoxicity, and a high capacity for drug encapsulation (Li & Mooney, 2016) and have thus been widely used in recent years for cancer treatment (Fang et al., 2018; Wu et al., 2019). Drugs are administered directly into the tumor or near to it through the hydrogel (Pan et al., 2018). A hydrophilic polymer-based crosslinked 3D network could be used to localize drugs. Drug toxicity is thus confined inside a localized area containing tumor cells. Meanwhile, the localized hydrogel can deliver the drugs continuously and efficiently to the tumor. To increase this characteristic, a range of systems with varying compositions have been developed, including PEG, PPZ, and PLGA (Bu et al., 2017; Gajendiran et al., 2019).

Hydrogels have gained interest as a depot for local drug delivery over the last decade, particularly in restricted and localized pathology. Based on amine aldehyde chemistry, a unique class of biocompatible and biodegradable adhesive hydrogels has been prepared (Wang et al., 2021). Aldehydes derived from dextran macromolecules provide adhesion via causing interaction with tissue amines, whereas unreacted aldehydes undergo reaction with dendrimer's amines to generate material cohesive bulk. Nonreactive dextran aldehyde crosslinks with dendrimer amines, lowering the free aldehyde content, preventing attachment to other organs, and forming the hydrogel's cohesive bulk. Researchers investigated how endogenous triggers alter the structure and characteristics of hydrogels in both *in vitro* and *in vivo* (Wang et al., 2021; Zhang et al., 2021). Additionally, research was also conducted regarding the possibility of using this structure as a nanoparticle depot for triggered cargo release (Deng et al., 2021; Li et al., 2022). A design of rational hydrogel that combines specific stimuli sensing which results *in situ* assembly of hydrogel, prompted hydrogel disintegration, and triggered release of drug from embedded cargo NPs, all of which can result in on-demand therapeutic effects. This section will describe our research on tissue- and disease-responsive dendrimer–dextran hydrogels and will provide insight into how to build materials that impart local therapeutic effects on-demand.

### 4.1. Thermosensitive hydrogels

**Figure 4.**
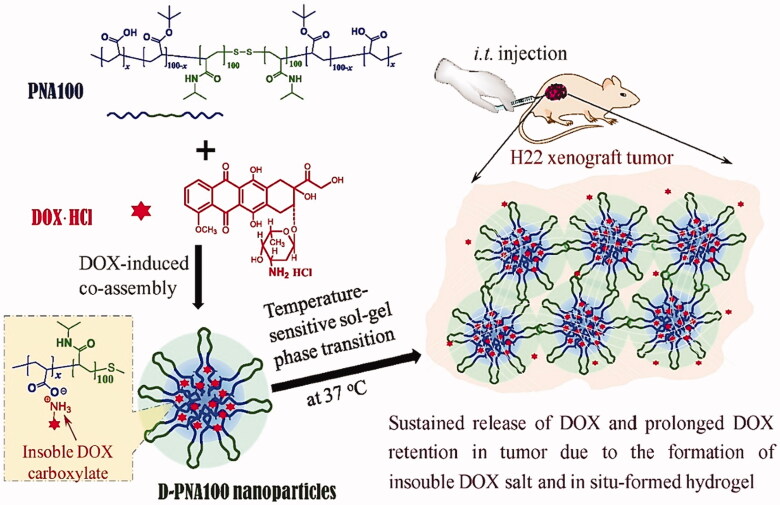
D-PNAx hydrogels for i.t. injection of targeted chemotherapy using DOX-induced co-assembling nanoparticles are shown schematically. Reproduced with permission from Wan et al. (2016).

Another stimuli-responsive hydrogel, thermosensitive hydrogels, have been developed most frequently in recent years. Due to a low LCST, over which gels transition occurs to a gel state while remaining in the solution form. Thus, a hydrogel with an LCST between body and room temperature is ideal for *in situ* gelations after injection. Numerous polymers with thermosensitive characteristics have been employed to date, including PNIPAM having the capability to undergo sol–gel transition at 32 °C of LCST or lower when hydrophobic groups are introduced (Yoshida et al., 1995). Elastin-like polypeptides (ELPs), synthetic polypeptides, have a controllable LCST ranging from 0 to 100 °C (Meyer & Chilkoti, 1999). In regards to thermo-responsive hydrogels, there has been an increase in interest in combining thermal sensitivity with other stimuli-responsive features in order to obtain more effective delivery, such as thermal/pH dual sensitive hydrogels and thermal-magnetic dual sensitive hydrogels (Khan et al., 2019; Nandwana et al., 2019; Ruan et al., 2019). Additionally, the loading of drugs in hydrogels has evolved from a single therapeutic agent to co-loading with other new therapeutic agents, most notably cytokines, antibodies, and genes (Lv et al., 2018; Zhao et al., 2019). In comparison to the past usage of exclusively *in situ* injections, administration routes have become more diverse, for example, the use of transdermal patches (Makhmalzadeh et al., 2018). Aside from the functional behavior of thermosensitive hydrogels, release kinetics was also investigated at various temperatures and the precise control of the release behavior was determined by altering the hydrogel porosity (Danyuo et al., 2019) (Figure 4).

Wan et al. (2016) described the co-assembly of DOX and nanomedicines (D-PNAx) to produce temperature-sensitive PNAx triblock polymers for localized liver cancer chemotherapy (Wan et al., 2016). Through intratumoral delivery of D-PNAx nanomedicines combined with thermal-sensitive *in situ* hydrogels formation, good anticancer efficacy against H22 tumors was demonstrated. By simply combining doxorubicin hydrochloride and PNAx polymers, D-PNAx nanomedicines displayed significant entrapment efficacy. The controlled release trend of D-PNA100 nanomedicines demonstrated only 9.4% release of DOX in a single day and 60% over 10 days. D-PNA100 nanomedicines demonstrated remarkable anticancer effectiveness against H22 tumors when administered intratumorally. The primary limitations of systemic chemotherapy for HCC are the low accumulation of the anticancer drug in tumors and their systemic toxicity. Thermoresponsive hydrogels are well-suited for the efficient local administration of chemotherapeutics at the same time. Gao et al. developed an *in situ* injectable hydrogel system with thermosensitive features for intratumoral administration of norcantharidin-loaded nanoparticles (NCTD-NPs) and DOX to HCC tumors (Gao et al., 2021). The synthesized drug-loaded hydrogel displayed excellent thermal sensitivity, remaining liquid at room temperature but immediately transforming into a non-flowing gel upon exposure to body temperature, and sustained the release of the drugs. *In vitro* experiments demonstrated that the hydrogel loaded with drugs was significantly more antiproliferative than the free drugs in HepG2 cells. Antitumor assays in an animal model have shown that the hydrogel effectively inhibited tumor growth, reduced adverse effects, and prolonged survival in mice carrying H22 tumors compared with other groups. Additionally, immunohistochemistry staining demonstrated that the drug-loaded hydrogel group had considerably lower levels of Ki-67 and CD31 expression than the other groups (*p* < .05), showing that the drug-loaded hydrogel successfully reduced angiogenesis and proliferation of tumors. The developed hybrid thermosensitive hydrogel with improved therapeutic efficacy and sustained drug release potential was shown to be a potential technique for the local-regional therapy of HCC by intratumoral injection. Peng et al. constructed embelin-loaded thermosensitive injectable hydrogel, i.e. Embelin/PECT^gel^ (Peng et al., 2014). Embelin/PECT^gel^ was investigated for its cytotoxicity and antitumor activity against liver cancer (*in vitro* and *in vivo*). The results suggested that Embelin/PECT^gel^ released the therapeutics continuously, resulting in enhanced toxicity for H22 cells upon comparison with free embelin. Within seconds of injection, the aqueous Embelin/PECT^gel^ solution transformed into a gel, which was later being degraded *in vivo*. A significant antitumor effect was observed for Embelin/PECT^gel^ at a dose of 0.5 mg per mouse comparable to the treatment of embelin solution delivered at 6 mg per mouse of the total dose. Hence, Embelin/PECT^gel^ can be considered a novel drug candidate for advanced HCC and represents a novel drug delivery system for the liver.

In one general aspect, US 2013/0022.545 A1 temperature-sensitive hydrogel was composed of block copolymer for the liver cancer treatment via interventional injection. The synthesized hydrogel was capable of hepatic arterial catheterization and a therapeutic agent (DOX) is loaded inside the drug delivery system. The hydrogel is in the sol state at room temperature and undergoes a phase transition into the gel state inside the hepatic artery, at body temperature, thereby delaying or blocking the blood supply of the hepatic artery, and slowly releasing the DOX during the phase transition into the gel state inside the hepatic artery under the body conditions of pH 7.0–7.4 and temperature 37 °C (Lee et al., 2013).

### 4.2. pH-sensitive hydrogels

**Figure 5.**
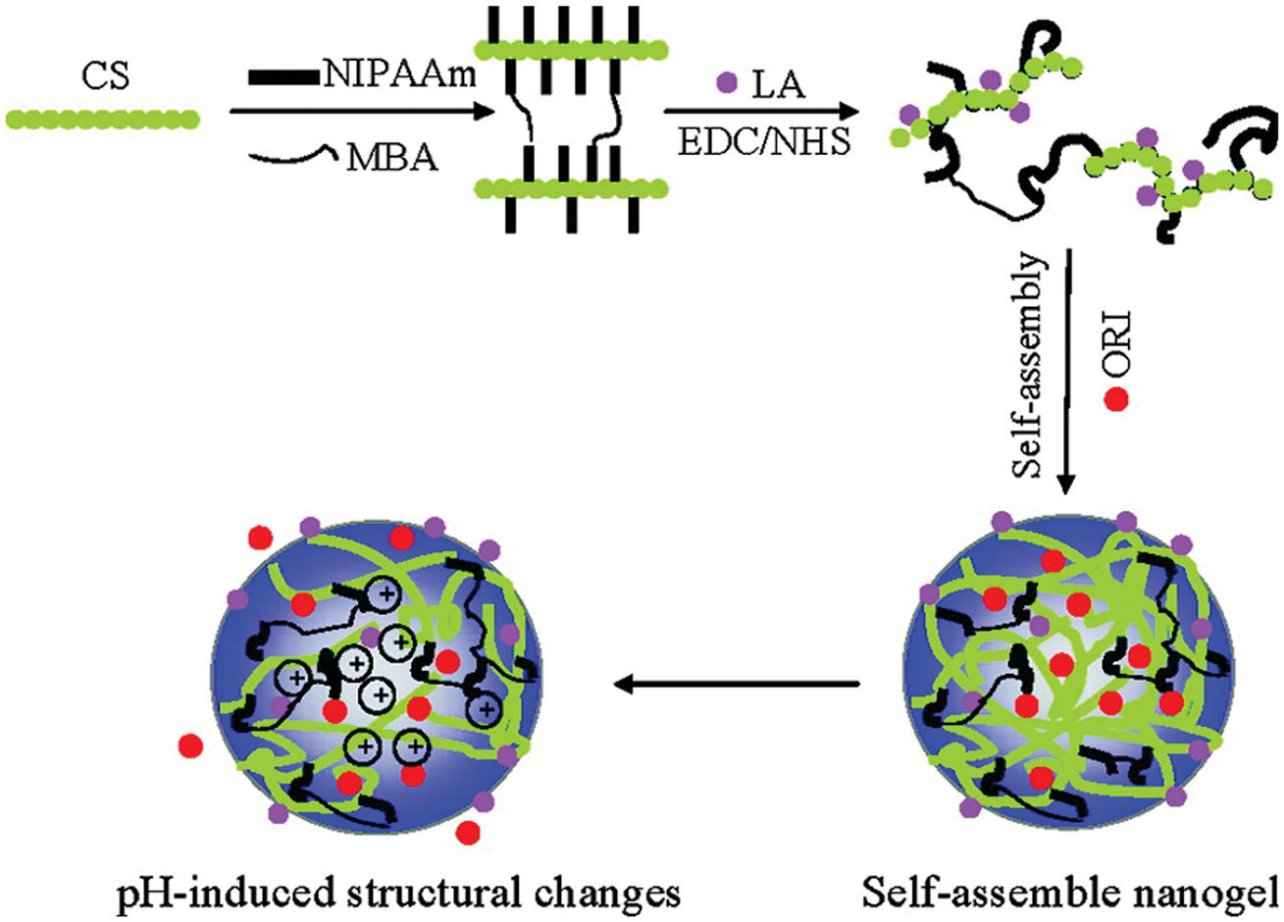
Schematic diagram depicting the concept of Gal-CS-g-PNIPAM nanogels. Reproduced with permission from American Chemical Society 2011 (Duan et al., 2011).

pH-responsive hydrogels have widely been used in various applications, including wound healing and antimicrobial therapies, as well as tumor therapy (Qu et al., 2018, 2019; Wu et al., 2019). The primary reason for developing pH-sensitive hydrogels includes the extracellular pH (pHex) of tumors (5.8 and 7.2), and endosomal or lysosomal pH is roughly 5.5, both of which are more acidic than the normal tissue pH (∼7.40) (Ojugo et al., 1999). Thus, both the extracellular and intracellular endosome environments produce an acidic environment favorable to hydrogel degradation and drug release (Eckmann et al., 2014). Protonation of carboxyl groups (e.g. ionizable groups) or hydrolysis of acid-labile bonds can be attributed to pH sensitivity. pH-sensitive hydrogels have emerged as promising options to deliver drugs to tumor tissue due to their biocompatibility, biodegradability, and potential to selectively release the drug in an acidic environment. In the domain of hydrogels with pH-sensitive characteristics, pH-sensitive hydrogel theranostics and prodrugs are becoming increasingly popular. Chemotherapy medicines are conjugated with hydrogel monomers via acid-labile linkages to produce the prodrugs. They have little pharmacological activity at physiological pH (∼7.40), but in an acidic environment, they transform into chemotherapeutic agents that kill tumor cells. Under acidic conditions, pH-sensitive theranostics release both pharmaceuticals and diagnostic agents concurrently from the pH-responsive hydrogel to fulfill the aim of integrating diagnosis and therapy (Figure 5).

In addition to reducing drug accumulation in normal tissue, pH-sensitive hydrogel prodrugs improve treatment efficacy by selectively releasing drugs at acidic tumor sites, reducing systemic harmful effects. Raza et al. reported the invention of a paclitaxel delivery for tumor targeting via pH-sensitive FER-8 peptide hydrogel (Raza et al., 2019). The loading ability, structure, sensitivity toward acid, rheology, drug release, morphology, cytotoxicity (*in vitro*), and *in vivo* efficacy of the pH-sensitive hydrogel system were determined in H22 tumor-bearing mice. *In vitro* cytotoxicity tests revealed that as compared to free pharmaceuticals, the FER-8 peptide hydrogel enhanced the accumulation of the drug in HepG2 cells and suppressed the HepG2 tumor growth efficiently. Additionally, *in vivo* investigations demonstrated that the FER-8 peptide hydrogel loaded with paclitaxel intensely boosted the drug content in tumor tissues and exhibited sustained retention for up to 96 hours at the tumor site. The anti-tumor studies *in vivo* verified HG-pH-sensitive PTX's capabilities, which enabled the drug to be released under the influence of acidic pH of tumor-bearing area, resulting in sustained drug release and increased tumor suppression. In conclusion, HG-PTX is an interesting method and prospective carrier for the efficient delivery of chemotherapeutics. Xu et al. reported the development of a biodegradable and pH-responsive DOX@ACC/PAA NP (pH 7.4–5.6) hydrogel via encapsulation of DOX in amorphous calcium carbonate (ACC) nanoparticles stabilized with poly(acrylic acid) (PAA) (Xu et al., 2019). DOX-loaded NPs are small (62 ± 10 nm) in size, have good stability in serum, have high drug encapsulation (>80%), and have high loading potential (>9%). The drug release behavior from NPs can also be adjusted to higher pH-responsive ranges (pH 7.7–6.0) by doping appropriate concentrations of Sr^2+^ or Mg^2+^, allowing drug delivery to certain tissues with a less acidic condition. Lower acute toxicity of drug and tumor inhibition is proven further using zebrafish models and intracellular uptake assays, and the particles further improve drug accumulation and pharmacokinetics in mice xenograft tumors, resulting in better tumor growth suppression. The current hybrid nanocarrier may find widespread applicability in tumor therapy due to its outstanding biodegradability, biocompatibility, and tunable drug release behavior.

Qu et al. established a series of pH-sensitive polysaccharide-based self-healing injectable hydrogels for the delivery of an anticancer agent to HCC (Qu et al., 2017). By utilizing the Schiff base formation with pH-responsive behavior, we demonstrated the potential of pH-responsive hydrogels based on N-carboxyethyl chitosan (CEC) and dibenzaldehyde-terminated PEGDA as a carrier for DOX delivery toward HCC therapy. They exhibited sufficient cytocompatibility to support L929 cell proliferation and were capable of releasing Dox to HepG2 cells. The degradation behavior of CEC/PEGDA hydrogels was evaluated in an acidic (PBS, pH = 5.5) and physiological (PBS, pH = 7.4) microenvironment. It was demonstrated unambiguously that samples in PBS with a pH of 5.5 degraded significantly more rapidly than pH of 7.4. After 48 hours, the CEC/PEGDA20 lost approximately 14% of its mass at pH 7.4 and approximately 40% of its mass at pH 5.5. Additionally, the components used to synthesize the hydrogels (CEC and PEG) are biodegradable or can be cleared by the renal tubules. Therefore, they can be completely degraded *in vivo* after exerting the function of a delivery vehicle. All of these findings indicate that these injectable self-healing hydrogels with pH-responsive behavior are excellent candidates for use as anticancer drug delivery carriers in the treatment of HCC.

### 4.3. Photosensitive hydrogels

**Figure 6.**
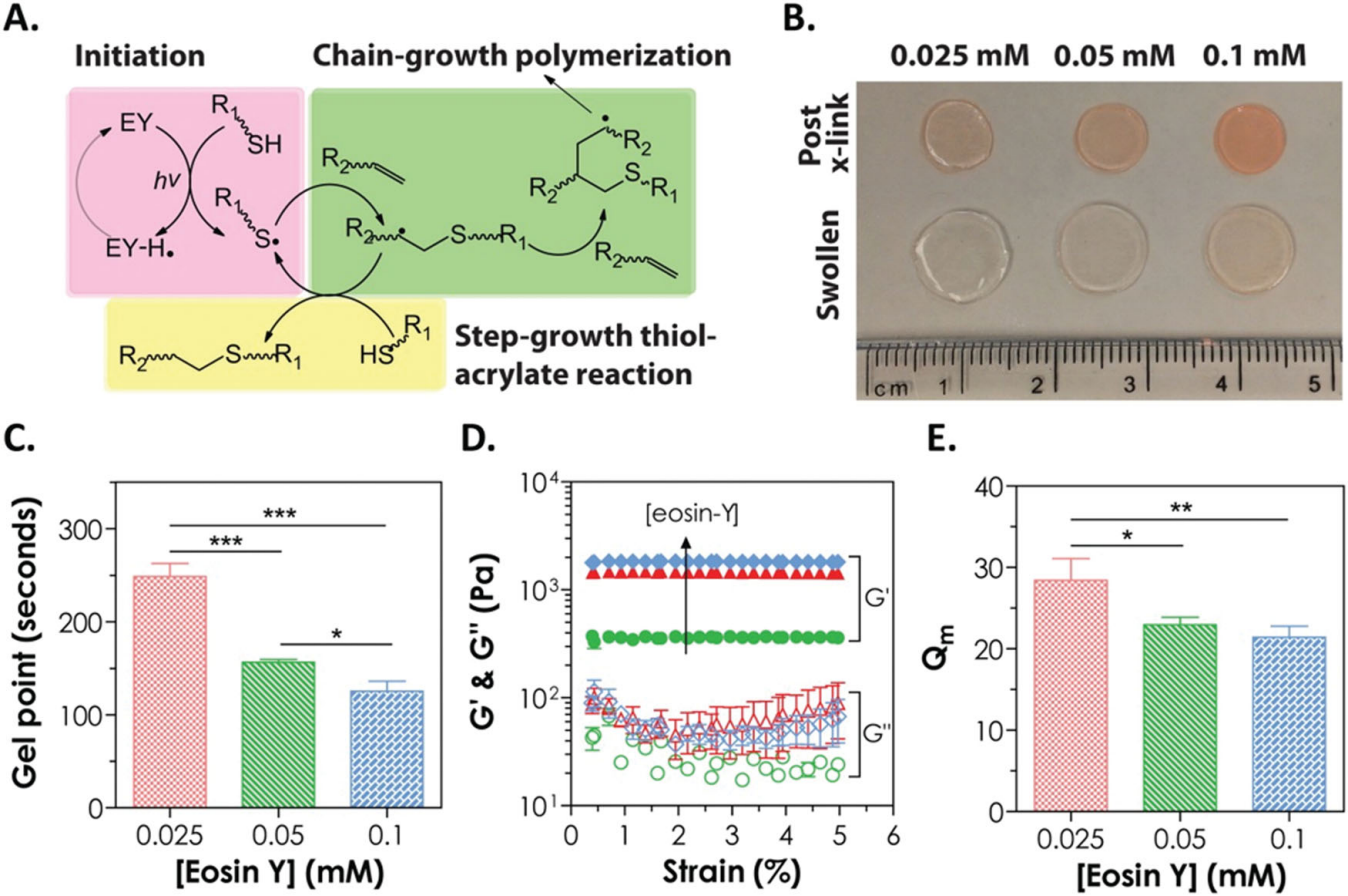
(A) Mechanism of visible light initiated mixed-mode step-chain-growth thiol-acrylate photopolymerization. (B–E) Physical properties of visible light-cured PEGDA thiol-acrylate hydrogels formed with 0.025, 0.05, or 0.1 mM of eosin-Y. (B) Photographs; (C) gel points; (D) elastic (*G*′) and viscous (*G*″) modulus, and (E) equilibrium mass swelling ratio (Qm). Reproduced with permission from Wiley Online Library (Hao & Lin, 2014).

Consider light as an external stimulus; it has been shown to increase not just hydrogel formation but also the release of loaded drugs. First, the changes occur in the hydrogel conformation upon exposure to ultraviolet, radiation, or visible light, resulting in a sol–gel transition and the production of hydrogels (e.g. free-radical polymerization, cis–trans isomerization of azobenzenes under the influence of UV light) (Lim et al., 2014; Pereira & Bártolo, 2014). Second, the hydrogel is loaded with photothermal conversion-efficient material and delivered into the body. During the exposure of gel to radiation (usually NIR), light energy is converted to heat energy to disturb the structure of gel and drug release and destroy the cells associated with the tumor. This treatment is referred to as photothermal therapy currently focusing on materials having enhanced efficacy toward photothermal conversion, such as metal and nonmetallic compounds, as well as organic dyes (Huang et al., 2008; Cheng et al., 2013). Drugs with controlled release and repeatable chemo-thermal therapy have both attracted public interest (Cheng et al., 2013). Due to its capacity to permeate living tissue, NIR irradiation is commonly used in photothermal therapy as an extrinsic stimulus (Zhang et al., 2012). Finding a material with high efficiency for photothermal conversion is critical in PTT. For the first time, GhavamiNejad et al. introduced dopamine nanoparticles (DPs) into a stimuli-responsive pNIPAAm-co-pAAm hydrogel as an anticancer drug and photothermal agent (GhavamiNejad et al., 2016). When the hydrogel was illuminated with NIR light, the DP nanoparticles induced hyperthermia around tumor regions by absorbing light, thus accelerating medication release and killing tumors (Figure 6).

Hao et al. developed a degradable PEGDA hydrogel system using simple macromer formulations and visible light-triggered thiol-acrylate photopolymerization (Hao & Lin, 2014). As a photo-sensitizer, Eosin-Y is included in this gelation system together with PEGDA, as well as a bi-functional thiol (dithiothreitol, DTT) that serves as a cross-linker and co-initiator and N-vinylpyrrolidone (NVP) as a co-monomer. Under the influence of intense visible light, gelation was accomplished utilizing a mixed-mode step-chain polymerization approach. This visible light-generated mixed-mode cross-linked hydrogel decomposed hydrolytically owing to the formation of thiol-ether-ester linkages as a result of thiol-acrylate interactions. The bifunctional thiol content of the prepolymer solution and hydrolytic degradation rate was discovered to have an exponential growth relationship. Finally, we used *in situ* encapsulation of Huh7 cells to test the cytocompatibility of these mixed-mode cross-linked degradable hydrogels. Huh7 cells that had been encapsulated remained alive and proliferated over time, forming cell clusters. The NVP addition at a greater dose (0.3%) did not affect Huh7 cell viability, but it did result in a decrease in metabolic activity of the cell, followed by an increase in urea secretion from the encapsulated cells. Anticancer photodynamic treatment using photosensitizer chemicals integrated into hydrogels has also been reported. For instance, phthalocyanine zinc (ZnPc) was utilized in the formation of a hybrid hydrogel including PEGDA, PEG 400, and phosphotungstic acid as both a photoinitiator and a photosensitizer (Wang et al., 2013). After being exposed to NIR laser light, ZnPc becomes excited and produces singlet oxygen (^1^O_2_), which causes a significant drop in HeLa cell viability and proliferation rates. Hydrogel also has a high degree of biocompatibility, making it ideal for incorporating anticancer drugs into a combination or synergic therapy (Wang et al., 2013). Similar results were reported more recently using a PEGDA-based hydrogel containing methylene blue-sensitive mesoporous titanium (IV) oxide (TiO_2_) nanocrystals produced *in situ* through NIR photopolymerization (Chang et al., 2019). TiO_2_ was employed as a photoinitiator and photosensitizer, while methylene blue was used as a photosensitive additive to enhance TiO_2_'s effects.

### 4.4. Ultrasound-sensitive hydrogels

Thermal and nonthermal effects of ultrasound on biological tissues are both present. Thermal effects are the conversion of acoustic energy to thermal energy, which increases tissue temperature, disruption of cell membranes, and increases vasculature permeability (Gao et al., 2005). While nonthermal effects, known as cavitation effects, are ultrasound-mediated tiny gas bubbles acting as microreactors, causing increased pressure and permeability of cell membrane, as well as the drug release received by cells (Manouras & Vamvakaki, 2017). Hydrogels with ultrasound sensitivity have typically been used in conjunction with other gene carriers or stimuli-responsive hydrogels for years (Chen & Du, 2013). They can also be used as a distinct anticancer drug carrier because of their deep permeation and visualization. Exploration of ultrasound-sensitive gels for simultaneous diagnosis and therapy is another active research area (Kumar & Han, 2017). Ultrasound has a variety of effects on the mechanism of delivery from gels. Acoustic vibrations can produce localized high heat, as evidenced by the recent use of high-intensity focused ultrasound (HIFU) in tumor treatment (Jeong et al., 2016). The release of antitumor drugs from thermosensitive hydrogels is also regulated by this thermal effect. Furthermore, ultrasound-induced cavitation greatly improves the permeability of cell membranes in ultrasound-sensitive hydrogel carriers.

**Figure 7.**
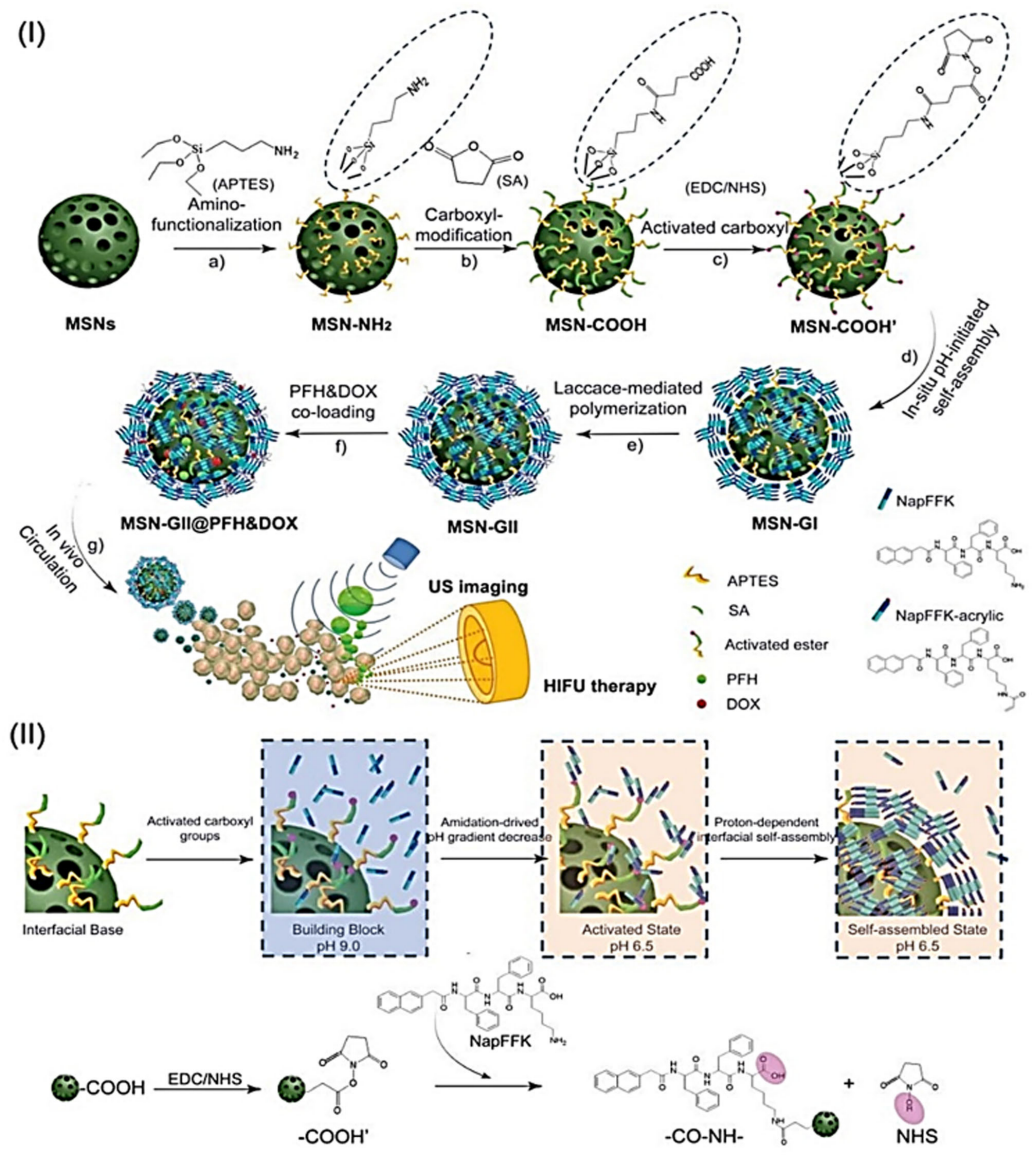
(I) Schematic illustration of MSN-GII@PFH&DOX includes (a) the MSNs surface modification with amino groups and (b, c) carboxyl groups activation, (d) *in situ* amidation-initiated self-assembled supramolecular nanogels on the interface of MSNs (MSN-GI), (e) ternary inorganic-supramolecular-polymeric nanogels (MSN-GII) by laccase-mediated polymerization, (3) co-loading of guest molecules PFH and DOX, and (4) ultrasound imaging and imaging-guided HIFU ablation of tumors. (II) Schematic illustration of the mechanism of *in situ* amidation-fueled self-assembly. Reproduced with permission from American Chemical Society 2019 (Wang et al., 2019).

Wang et al. reported an *in situ* amidation-driven oligopeptides self-assembly and subsequent enzymatic cross-linking to prepare ternary inorganic-supramolecular-polymeric nanogels based on mesoporous silica nanoparticles (MSN) as multifunctional theranostic agent for combined ultrasound imaging and imaging-guided HIFU therapy (Wang et al., 2019). Supramolecular hybrid nanogels (MSN-GI) were first synthesized *in situ* via an amidation reaction on the surface of peptide gelators (NapFFK, NapFFK-acrylic). Following that, redox-responsive supramolecular-polymeric hybrid nanogels (MSN-GII) were synthesized via radical postpolymerization of vinyl peptide gelators with monomer and disulfide cross-linker using laccase. After therapeutic loading and guest molecules with ultrasound sensitivity, the multifunctional theranostic agent (MSN-GII@PFH&DOX) was finally constructed. This supramolecular-polymeric hybrid nanogel system can be used as a potential theranostic agent for efficient tumor-responsive drug release, enhanced ultrasound contrast visualization, and enhanced imaging-guided HIFU therapy, both *in vitro* and *in vivo* (Figure 7).

### 4.5. Magnetic responsive hydrogel

Aside from the most frequent stimuli for the production of sensitive hydrogels, such as temperature, pH, and light, other parameters such as a magnetic field or ionic strength can also alter the physicochemical properties of stimuli-responsive hydrogels.

Hydrogels containing iron oxide nanoparticles with paramagnetic properties are commonly used to make magnetic-sensitive hydrogels. As they vibrate upon exposure to the magnetic field, which results in increased local temperature by thermal ablation mechanisms, promote therapeutic efficacy. Additionally, these systems are frequently associated with thermosensitive hydrogels in which an increase in temperature results in drug release, promoting synergistic cytotoxicity via thermal and chemotherapeutics. As with NIR, this strategy exhibits spatial and temporal activity while being minimally invasive and capable of penetrating deep tissue (Kasiński et al., 2020).

**Figure 8.**
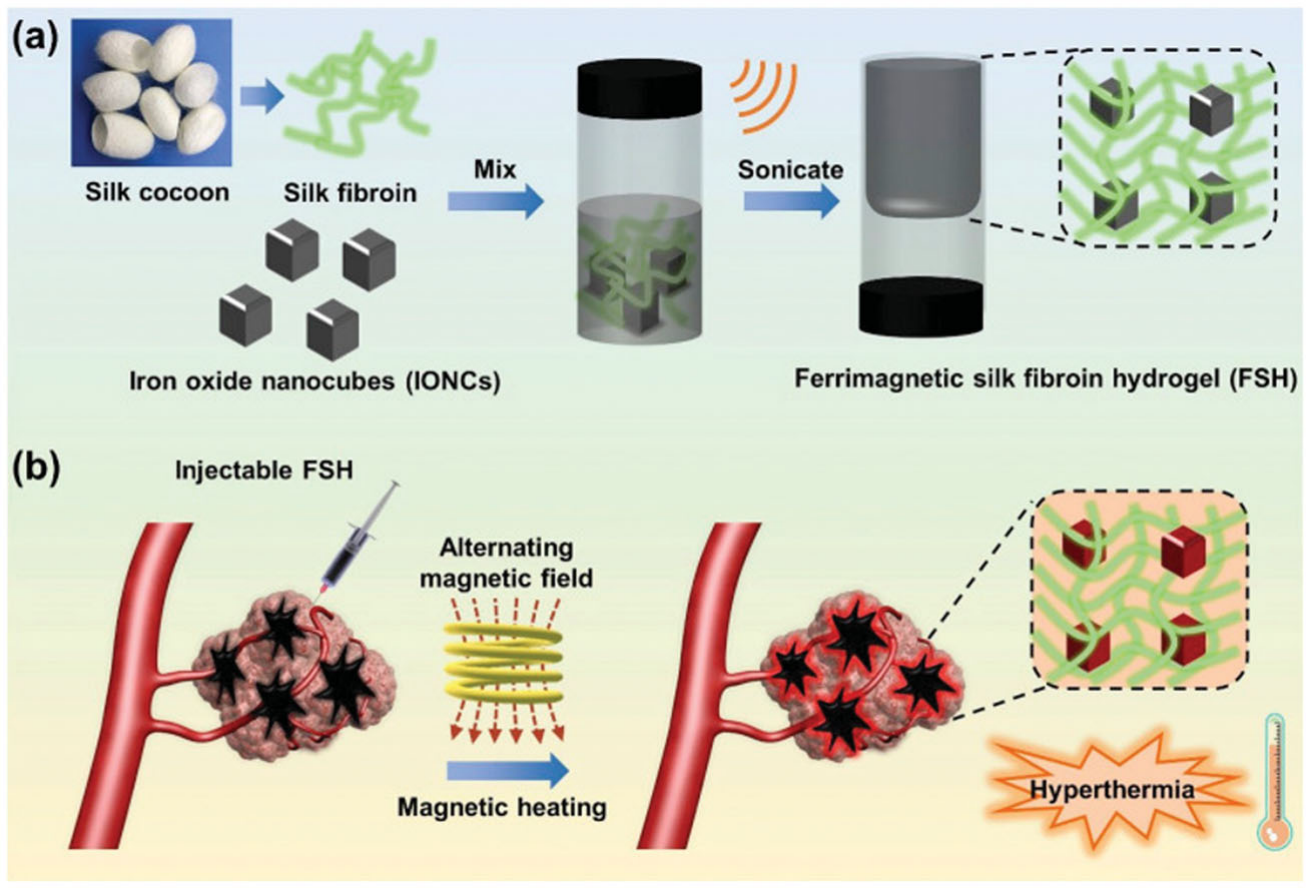
(a) Schematic illustration of the preparation of ferrimagnetic silk fibroin hydrogel (FSH); (b) injectable FSH guided magnetic hyperthermia therapy of tumor under alternating magnetic field (AMF). Reproduced with permission from reference (Qian et al., 2020).

Cisplatin was combined with magnetic Fe_3_O_4_ NPs (MNPs) and physically embedded into the hydrogel matrix following gelation to develop a drug-loaded magnetic nanocomposite (P(NIPAM-*co*-DMA)/Fe_3_O_4_) (Salimi et al., 2018). Upon exposure to an external magnetic field, the synthesized nanocomposite dispersion was found to be transparent, demonstrating the super magnetic behavior of the nanocomposite. The highest release behavior was observed at pH 5.8, 40 °C with augmented efficacy in HepG2 cells owing to improved and targeted delivery. In another work, Fe_3_O_4_ NPs and CTS were successfully utilized for the synthesis of methotrexate (MTX)-encapsulated magnetic-/pH-responsive hydrogel beads through a one-step gelation process (Wu et al., 2016). Additionally, the hydrogel beads were found to be extremely sensitive to pH changes as well as having an extremely homogeneous porous structure with super magnetic potential. The hydrogel beads released the encapsulated MTX at pH 4.0 HepG2 cell proliferation was effectively inhibited by MTX-encapsulated magnetic hydrogel beads in the WST-1 test. All of these findings suggest that pH-sensitive magnetic CTS hydrogel beads encapsulated with MTX may be a promising vehicle for targeted cancer treatment. Yan et al. reported the *in situ* formed magnetic hydrogel using magnetite decorated reduced graphene oxide nanosheets (denoted as FG) and NDP hydrogel with strong adhesion in wet conditions, promising thermal responsiveness for use in biomedical applications, high magnetic hyperthermia, and biocompatibility, leading to efficient HCC multidisciplinary treatment including postoperative treatment and transarterial embolization therapy (Yan et al., 2022). The hydrogel's ability to reduce postoperative recurrence was demonstrated *in vivo.* Further evidence of the thermal-responsive hydrogel's hemostatic properties was provided by studies using the liver scratch model and resection of a liver tumor. CT imaging showed the complete embolization of the arterial vessels of a rabbit liver tumor during a vascular intervention operation, suggesting that it could be used as a multidisciplinary responsive material to an external magnetic field for the treatment of HCC. Qian et al. synthesized an injectable magnetic hydrogel based on iron oxide nanocubes (IONCs) and silk fibroin (Qian et al., 2020). Using ultrasound-guided interventional treatment, it was discovered that the manufactured hydrogel may be injected into tumors, particularly rabbit HCC in deeper regions. This hydrogel, unlike photothermal agents, has remote hyperthermia capability in an alternate magnetic field, which resulted in effective magnetic hyperthermia of deep tumors including a subcutaneously implanted tumor model in Balb/c mice after covering it with fresh pork tissue and an orthotopic liver tumor transplantation in rabbit. Additionally, by incorporating IONCs in a silk fibroin hydrogel, undesirable thermal damage can be avoided, as compared to direct delivery of monodispersed magnetic nanoparticles (Figure 8) (Table 2).

**Table 2.** List of stimuli-responsive hydrogel for liver cancer therapy.

Stimulus	Hydrogel	Therapeutic agent	Delivery rout	Cell line (*in vitro*)	Tumor model (*in vivo*)	Ref.
Temperature	Hydrogels of DOX-induced co-assembling nanoparticles (D-PNAx)	Doxorubicin	Intratumoral delivery	–	H22 tumor-bearing mice	Wan et al. (2016)
Temperature	Pluronic F127 (PF127) based hydrogel	Doxorubicin	Intratumoral delivery	HepG2 cells	H22 tumor-bearing mice	Gao et al. (2021)
Temperature	Embelin/PECT^gel^	Embelin	Peritumoral injection	H22 cells	Hepatic cancer mouse models	Peng et al. (2014)
Temperature	Hydrogel composed of block copolymer	Doxorubicin	Interventional injection	–	Rabbit with VX2 tumor	Lee et al. (2013)
pH	FER-8 peptide hydrogel	Paclitaxel	Intratumoral injection	–	H22 tumor-bearing mice	Raza et al. (2019)
pH	DOX@ACC/PAA NP	Doxorubicin	Intravenous injection	SMCC-7721 cells	SMCC-7721 xenografts zebrafish, tumor-bearing mice	Xu et al. (2019)
pH	Hydrogels based on CEC and PEGDA	Doxorubicin	Subcutaneous injection	HepG2 cell	–	Qu et al. (2017)
Light	Hydrogel of PEGDA	Eosin-Y	–	Huh7 cells	–	Hao & Lin (2014)
Light	Hydrogel of PEGDA, PEG 400, and phosphotungstic acid	Phthalocyanine zinc	–	HeLa cell	–	Wang et al. (2013)
Ultrasound	Supramolecular hybrid nanogels (MSN-GI)	Doxorubicin	Postintravenous injection	SMMC-7721 cells	Rabbits bearing a VX2 liver tumor	Wang et al. (2019)
Magnet	[P(NIPAM-*co*-DMA)/Fe_3_O_4_]	Cisplatin	–	HepG2 cells	–	Salimi et al. (2018)
Magnet	Magnetic-/pH-responsive hydrogel beads	Methotrexate	–	HepG2 cells	–	Wu et al. (2016)
Magnet	NDP-FG hydrogel	–	Locally administrated at the surgical site	–	Balb/c nude mice	Yan et al. (2022)
Magnet	Magnetic hydrogel based on silk fibroin and iron oxide nanocubes	–	Intratumoral injection	–	VX2 liver tumor-bearing rabbits and Balb/c mouse bearing subcutaneous tumor	Qian et al. (2020)

## 5. Active targeting

Active targeting can be used to deliver therapeutic drugs to tumors with high specificity and efficiency using various ligands that serve as a targeting moiety (Wang et al., 2019). It is possible to incorporate active targeting into nanomedicines because of their large surface area. Active targeting may be able to overcome passive targeting's limitations (Yan et al., 2015). It is possible to include active targeting capabilities in NPs because of their large surface area and sophisticated chemical ligation procedures. This targeting technique is based on ligand interactions with receptors or antigens that are overexpressed on the target cells resulting in receptor-mediated endocytosis and the selective delivery of NPs (Rosenblum et al., 2018). Numerous nanomedicines based on an active targeting approach have been studied for liver cancer therapy, with several demonstrating increased anticancer activity while avoiding side effects (Varshosaz & Farzan, 2015; Lu et al., 2018). Currently, the most often employed ligands in liver cancer therapy include galactose, glycyrrhetinic acid, folate, and short peptides, as well as macromolecular ligands such as transferrin (Tf), aptamers, antibodies.

Chen et al. replaced GA with naphthylacetic acid to form a supramolecular pro-gelator of curcumin modified by GA (GA-Cur) and a control compound Nap-Cur (Nap) (Chen et al., 2017). Both compounds were water-soluble and could form gels *in vitro* via the reduction of disulfide bond induced by glutathione (GSH). In buffer solutions, both gels were capable to give extended-release of Cur. We also used an MTT assay to determine the pro-gelators cytotoxicity to HepG2 cells, as well as LC-MS and fluorescence microscopy to determine their cellular uptake behaviors. As GA receptor overexpressed on the cancer cells of the liver, our GA-Cur pro-gelator demonstrated improved inhibition and cellular uptake through tumor cells of the liver compared to Nap-Cur. Hence, the GA-Cur has the potential to considerably inhibit HepG2 cell growth and found to be a novel nanosystem for the treatment of liver tumors. Furthermore, self-assembled hydrogel nanoparticles were developed by replacing the carboxymethylated (CM)-curdlan with sulfonylurea (SU) which was then functionalized with lactobionic acid for enhanced interaction with HepG2 cells. Ligand–receptor interactions with fluorescence-labeled LBA/CM-curdlan/SU hydrogel nanoparticles strongly illuminated HepG2. Other control cases did not exhibit luminescence. The authors conclude that hydrogel nanoparticles based on LBA/CM-curdlan/SU can be served as an effective approach for cancer therapy of the liver, owing to the immunostimulatory properties of CM-curdlan, the specific interactions mediated by the ligand-receptor, and the controlled release of the anticancer drug (Na et al., 2000).

## 6. Combination therapy

The development of a combination therapy-based platform is critical for enhanced antitumor efficacy and decreased recurrence of the tumor, as it enables the selective delivery of heat and anticancer drugs to the tumor site in a controlled manner. To achieve improved local tumor control, an injectable nanocomposite hydrogel was incorporated with PEGylated gold nanorods (GNRs) and CTS polymeric micelles loaded with PTX (PTX-M) were developed (Zhang et al., 2016). The thermosensitive hydrogel matrix can deliver and immobilize GNRs and PTX-M to the tumor after intratumoral injection. By using laser irradiation, the photothermal damage caused by the GNR is restricted to the tumor, while the healthy cells remained protected. PTX-M and the sustained drug release from the co-delivered PTX-M work together to destroy residual tumor cells. In Heps-bearing mice, this combination therapy shows superior benefits in suppressing tumor recurrence and extending survival as compared to photothermal therapy alone. Zhang et al. developed a sustained and programmed PCL-PEG-PCL thermosensitive hydrogel-based drug delivery system that was combined with multiwalled carbon nanotubes (CNTs) using rhodamine B (RB) and doxorubicin (DOX) as model drugs (Wei et al., 2018). In contrast to other dual drug delivery systems, *in vivo* fluorescence imaging can track co-loaded dual drug delivery systems in real-time due to their suitable fluorescence. Carbon nanotubes loaded with DOX were combined with hydrogels to develop a hydrogel-CNT system that can deliver drugs in a controlled manner. Nude mice were used as a model to track the real-time delivery of two drugs via florescence imaging, which was programmed to be delivered sequentially. Using two drugs in combination, the CNTs/hydrogel system has the potential to be used as a delivery vehicle with controlled released. The unique fluorescence signals of the two drugs can be used to track their delivery and distribution in the drug delivery system. On-demand combination chemotherapy may be guided by fluorescence imaging tracking *in vivo.*

**Figure 9.**
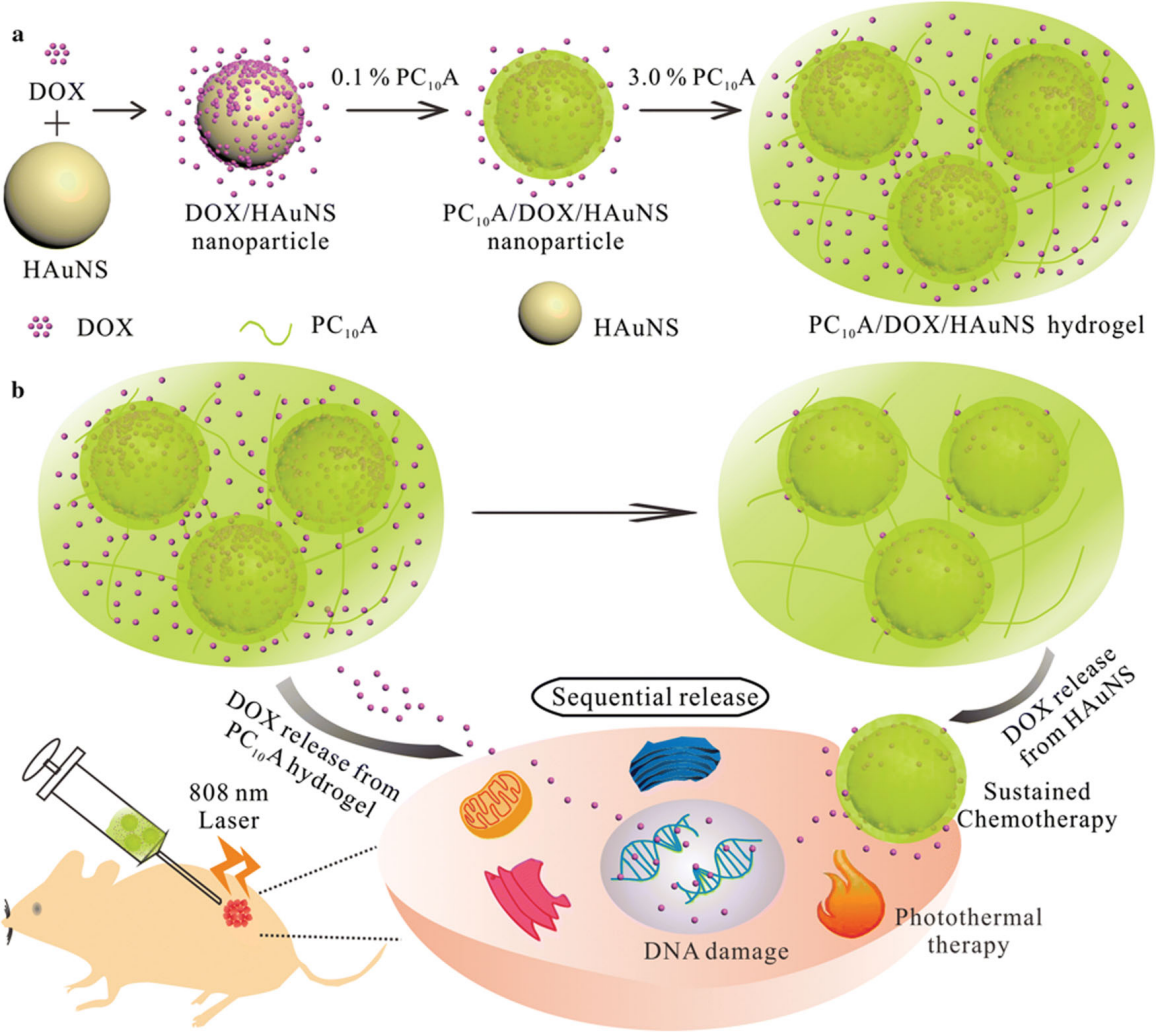
(a) In this schematic, we show the steps involved in developing PC_10_A/DOX/HAuNS hydrogels and nanoparticles, as well as the steps involved in dissolving the nanoparticles in the PC10A hydrogel. (b) The sequential drug release of PC_10_A/DOX/HAuNS hydrogels was used for long-term chemotherapy and photothermal therapy *in vivo* (Jin et al., 2019).

Jin et al. synthesized hollow gold nanoshells (HAuNS) and genetically engineered polypeptide-based injectable hydrogel for chemo-photothermal therapy of HepG2 tumor (Jin et al., 2019). We began by layer-by-layer coating with DOX and PC10A to develop a drug-loaded hybrid PC_10_A/DOX/HAuNS nanogel which were dissolved in PC_10_A hydrogel to develop injectable PC_10_A/DOX/HAuNS hydrogel with a variety of excellent properties. HAuNS and PC_10_A/DOX/HAuNS hydrogels were combined to release the drug DOX sequentially, with one part of the drug absorbed on the HAuNS and the other in the PC_10_A hydrogel. An impressively enhanced tumor inhibition was observed in both cell culture and mice models when the combined therapy of chemotherapy and photothermal therapy was used instead of either therapy alone. Due to this, the PC_10_A/DOX/HAuNS hydrogel has the potential to offer a new strategy for chemo-photothermal therapy (Figure 9).

Peng et al. synthesized a chemotherapeutic drug (liposomal DOX) and therapeutic radionuclide (^188^Re-Tin colloid) containing thermosensitive hydrogel (Peng et al., 2013). Temperature changes cause a sol–gel phase transition in the thermosensitive PCL-PEG-PCL copolymer, which quickly forms a gel at body temperature while remaining liquid at room temperature. The hydrogel was slow to release radionuclide and DOX, and the system remained stable for almost 10 days. After intra-tumor injection of Lipo-Dox/^188^Re-Tin hydrogel in HCC bearing mice, the tumor's retention, distribution, and therapeutic effect were investigated. 188Re-Tin loaded hydrogel had a significantly longer residence time in the tumor than Na 188Re perrhenate (Na 188ReO4). When the hydrogel undergoes thermal transition, the radionuclide ^188^Re perrhenate (^188^ReO4) quickly diffused from the tumor. Compared to treatment with either 188Re-Tin hydrogel or Lipo-Dox hydrogel, treatment with Lipo-Dox/_188_Re-Tin hydrogel significantly reduced tumor growth (up to 80% regression of well-established tumors on day 32). It is possible that this biodegradable injectable hydrogel would therefore allow for more precise localization of radiotherapy and chemotherapy for the treatment of HCC (Table 3).

**Table 3.** Hydrogel-based active targeting and combination therapy for liver cancer therapy.

Strategy	Hydrogel	Therapeutic agent	Delivery rout	Cell line (*in vitro*)	Tumor model (*in vivo*)	Ref.
Active targeting	Glycyrrhetinic acid (GA) modified curcumin supramolecular pro-gelator (GA-Cur)	Curcumin	–	HepG2 cells	–	Chen et al. (2017)
Active targeting	The hydrogel of lactobionic acid conjugated carboxymethylated (CM)-curdlan	Retinoic acid	–	HepG2	–	Na et al. (2000)
Combination therapy	The hydrogel of PEGylated gold nanorods (GNRs) and paclitaxel-loaded chitosan polymeric micelles (PTX-M)	Paclitaxel	Intratumorally injected	HepG2 cells	Heps-bearing mice	Zhang et al. (2016)
Combination therapy	PCL-PEG-PCL hydrogel combined with multiwalled carbon nanotubes (CNTs)	Doxorubicin and rhodamine B	Subcutaneous injection	BEL-7402	Mice	Wei et al. (2018)
Combination therapy	The hydrogel of genetically engineered polypeptide and hollow gold nanoshells (HAuNS)	Doxorubicin	Intratumoral injection	HepG2 cells	HepG2 tumor-bearing mice	Jin et al. (2019)
Combination therapy	The hydrogel of PCL-PEG-PCL copolymer	Doxorubicin	Intratumoral injection	BNL-Luc cell line	BNL-Luc tumor-bearing mice	Peng et al. (2013)

## 7. Limitations of hydrogel-based drug delivery systems in cancer treatment

Despite their numerous advantages, hydrogels do have a few limitations. Several hydrogels cannot be used in load-bearing applications due to their low tensile strength. These hydrogels prematurely dissolve or flow away from a designated site (Bai et al., 2020). This constraint may be more relevant in many conventional drug delivery applications (e.g. subcutaneous injection, *in situ* injection, and intratumoral injection). Perhaps a more significant concern is about the drug delivery capabilities of hydrogels. Drug loading into hydrogels may be limited in terms of amount and uniformity, particularly for hydrophobic drugs. The large pore diameters and high-water content of most hydrogels result in the rapid release of payloads, or burst release (Tsirigotis-Maniecka et al., 2021). The ease of application is also a significant concern. Some hydrogels can be injected, but many cannot be, so they have to be implanted through surgery. Due to these limitations, the use of hydrogel-based therapies in clinical applications may be restricted (Table 4).

**Table 4.** Advantages and shortcomings of different hydrogel delivery routes for cancer treatment.

Delivery route	Advantages	Shortcomings	Ref.
Oral delivery	Convenient administration	Harsh gastrointestinal tract environment, first-pass elimination	Minhas et al. (2018)
Transdermal delivery	No first-pass elimination, accurate delivery, convenient administration	Unknown pharmacokinetics	Zhao et al. (2018)
Intravenous injection	High absorption	Rapid renal clearance	Wu et al. (2019)
Pulmonary delivery	No first-pass elimination, high absorption	Strict size requirements	Zhu et al. (2017)
*In situ* implantation	No first-pass elimination, accurate delivery, cell therapy	Surgery needed	Smith et al. (2017)
*In situ* injection	No first-pass elimination, accurate delivery, self-assemble	Unknown pharmacokinetics	Yu et al. (2018)
Transarterial chemoembolization	Low systemic toxicity, selective administration	Complex preparation, X-ray needed	Ashrafi et al. (2017)

Furthermore, hydrogels prospective therapeutic application in cancer treatment is still in its inception. There are several reasons behind this (Cao et al., 2019). The tissue penetration depth of NIR is limited to 3 mm when employing 810 nm NIR of less than 1 W, limiting its use in treating subcutaneous tumors or skin cancer (Nakamura et al., 2019). Colorectal cancer and ovarian cancer are two examples of deep tumors in the human body that appear to be difficult to treat with this method. Photo-crosslinking hydrogels for shallow visible light penetration have the same issue. Even though many studies use subcutaneous tumors as an *in vivo* model, there are often important and complex anatomical structures (e.g. lymphatic systems, veins, arteries, and nerves) located around the tumor that can be eroded by carcinoma tissue and must be taken into account when developing a treatment plan for cancer (Nagakawa et al., 2020). There is also a lack of hydrogel-related research on degradation and metabolism, immunological response, and biodistribution in the body for patients of different sexes and ages. As a result, hydrogel-based cancer treatment has a long way to go before it can be practiced in clinics.

## 8. Conclusions and prospects

Hydrogel-based drug delivery systems can be used to treat many diseases, including cancer. Hydrogels water-loving nature and capability to swell and shrink under the influence of environmental signs or just the water presence make them appealing for drug delivery. They are highly porous, and the polymers used to make them can further be cross-linked to varying degrees. With a flexible physical structure, hydrogels can be used for more than just targeted drug delivery. They are used in tissue engineering, contact lenses, wound care, and hygiene. Hydrogels have made tremendous strides in targeted and localized drug delivery. They are modified with polymers of various types and targeting ligands, giving them unique drug delivery properties. A perfect hydrogel material should have a high biodegradability and not produce any toxic substances. Flexible stimulus responsiveness, and even multi-stimulus responsiveness, as well as high drug loading efficiency, are all desirable properties of an ideal hydrogel-based drug delivery system. In the context of cancer chemotherapy, *in situ* hydrogels not only act as loading drugs and drug carriers for unstable and poorly soluble drugs but also act as a localized depot for the subsequent release of multiple drugs. Nanogels have better biocompatibility and solubility than other nanoparticles and can avoid mononuclear phagocyte system clearance after chemical modification. Nanogels have a larger surface area and thus respond faster to a stimulus after *in situ* injections. Despite this, there are still challenges in translating experimental findings into clinical practice. As a result of the difficulty in detecting nucleic acids and therapeutic proteins, the metabolism and release rate of the hydrogels remain ambiguous *in vivo*. Also, while animal models can assess short-term biocompatibility, long-term biocompatibility is not guaranteed, specifically for the synthesized materials. Hence, rigorous evaluations of newly emerged materials should be considered. Comprehensive research is required to advance hydrogel-based delivery vehicles into clinical trials. The trend points to the use of precisely tunable and highly controlled hydrogels in the future. Interdisciplinary researchers should work together to make hydrogels a safe and practical chemotherapy approach.

## References

[CIT0001] Abdel-Halim E, Al-Deyab SS. (2014). Preparation of poly (acrylic acid)/starch hydrogel and its application for cadmium ion removal from aqueous solutions. React Funct Polym 75:1457–8.

[CIT0003] An B, Lee H, Lee S, et al. (2015). Determining the selectivity of divalent metal cations for the carboxyl group of alginate hydrogel beads during competitive sorption. J Hazard Mater 298:11–8. 25988716 10.1016/j.jhazmat.2015.05.005

[CIT0004] Andrade F, Roca-Melendres MM, Durán-Lara EF, et al. (2021). Stimuli-responsive hydrogels for cancer treatment: the role of pH, light, ionic strength and magnetic field. Cancers 13:1164. 33803133 10.3390/cancers13051164 PMC7963181

[CIT0005] Ashrafi K, Tang Y, Britton H, et al. (2017). Characterization of a novel intrinsically radiopaque drug-eluting bead for image-guided therapy: DC Bead LUMI™. J Control Release 250:36–47. 28188808 10.1016/j.jconrel.2017.02.001 PMC5416940

[CIT0006] Bai S, Zhang X, Lv X, et al. (2020). Bioinspired mineral–organic bone adhesives for stable fracture fixation and accelerated bone regeneration. Adv Funct Mater 30:1908381.

[CIT0007] Bair RJ, Bair E, Viswanathan AN. (2015). A radiopaque polymer hydrogel used as a fiducial marker in gynecologic-cancer patients receiving brachytherapy. Brachytherapy 14:876–80. 26481393 10.1016/j.brachy.2015.08.008 PMC4914602

[CIT0008] Barroso N, Guaresti O, Pérez-Álvarez L, et al. (2019). Self-healable hyaluronic acid/chitosan polyelectrolyte complex hydrogels and multilayers. Eur Polym J 120:109268.

[CIT0009] Bu Y, Shen H, Yang F, et al. (2017). Construction of tough, in situ forming double-network hydrogels with good biocompatibility. ACS Appl Mater Interfaces 9:2205–12. 28029238 10.1021/acsami.6b15364

[CIT0010] Bykov VJN, Eriksson SE, Bianchi J, et al. (2018). Targeting mutant p53 for efficient cancer therapy. Nat Rev Cancer 18:89–102. 29242642 10.1038/nrc.2017.109

[CIT0011] Caló E, Khutoryanskiy VV. (2015). Biomedical applications of hydrogels: a review of patents and commercial products. Eur Polym J 65:252–67.

[CIT0012] Cao J, Zhu B, Zheng K, et al. (2019). Recent progress in NIR-II contrast agent for biological imaging. Front Bioeng Biotechnol 7:487. 32083067 10.3389/fbioe.2019.00487 PMC7002322

[CIT0013] Center MM, Jemal A. (2011). International trends in liver cancer incidence rates. Cancer Epidemiol Biomarkers Prev 20:2362–8. 21921256 10.1158/1055-9965.EPI-11-0643

[CIT0014] Chai Q, Jiao Y, Yu X. (2017). Hydrogels for biomedical applications: their characteristics and the mechanisms behind them. Gels 3:6. 30920503 10.3390/gels3010006 PMC6318667

[CIT0015] Chang G, Zhang H, Li S, et al. (2019). Effective photodynamic therapy of polymer hydrogel on tumor cells prepared using methylene blue sensitized mesoporous titania nanocrystal. Mater Sci Eng C Mater Biol Appl 99:1392–8. 30889673 10.1016/j.msec.2019.02.056

[CIT0016] Chen G, Li J, Cai Y, et al. (2017). A glycyrrhetinic acid-modified curcumin supramolecular hydrogel for liver tumor targeting therapy. Sci Rep 7:44210–8. 28281678 10.1038/srep44210 PMC5345068

[CIT0017] Chen W, Du J. (2013). Ultrasound and pH dually responsive polymer vesicles for anticancer drug delivery. Sci Rep 3:2162–9. 23831819 10.1038/srep02162 PMC3703610

[CIT0018] Cheng L, He W, Gong H, et al. (2013). PEGylated micelle nanoparticles encapsulating a non‐fluorescent near‐infrared organic dye as a safe and highly‐effective photothermal agent for in vivo cancer therapy. Adv Funct Mater 23:5893–902.

[CIT0019] Cho EJ, Sun B, Doh K-O, et al. (2015). Intraperitoneal delivery of platinum with in-situ crosslinkable hyaluronic acid gel for local therapy of ovarian cancer. Biomaterials 37:312–9. 25453960 10.1016/j.biomaterials.2014.10.039 PMC4312226

[CIT0020] Choi JR, Yong KW, Choi JY, et al. (2019). Recent advances in photo-crosslinkable hydrogels for biomedical applications. BioTechniques 66:40–53. 30730212 10.2144/btn-2018-0083

[CIT0021] Danhier F, Feron O, Préat V. (2010). To exploit the tumor microenvironment: passive and active tumor targeting of nanocarriers for anti-cancer drug delivery. J Control Release 148:135–46. 20797419 10.1016/j.jconrel.2010.08.027

[CIT0022] Danyuo Y, Ani CJ, Salifu AA, et al. (2019). Anomalous release kinetics of prodigiosin from poly-N-isopropyl-acrylamide based hydrogels for the treatment of triple negative breast cancer. Sci Rep 9:1–14. 30846795 10.1038/s41598-019-39578-4 PMC6405774

[CIT0023] De Souza R, Zahedi P, Allen CJ, et al. (2010). Polymeric drug delivery systems for localized cancer chemotherapy. Drug Deliv 17:365–75. 20429844 10.3109/10717541003762854

[CIT0024] Deng J, Xu W, Wang J, et al. (2021). Cancer cell membrane-coated nanogels as redox/pH dual-responsive drug carrier for tumor-targeted therapy. J Mater Chem B. DOI:10.1039/D1TB00788B. 34486010

[CIT0025] Ding J, Li Q, Xu X, et al. (2018). A wheat straw cellulose-based hydrogel for Cu (II) removal and preparation copper nanocomposite for reductive degradation of chloramphenicol. Carbohydr Polym 190:12–22. 29628228 10.1016/j.carbpol.2018.02.032

[CIT0026] Duan C, Gao J, Zhang D, et al. (2011). Galactose-decorated pH-responsive nanogels for hepatoma-targeted delivery of oridonin. Biomacromolecules 12:4335–43. 22077387 10.1021/bm201270m

[CIT0027] Eckmann DM, Composto RJ, Tsourkas A, et al. (2014). Nanogel carrier design for targeted drug delivery. J Mater Chem B 2:8085–97. 25485112 10.1039/C4TB01141D PMC4251498

[CIT0028] El-Serag HB, Marrero JA, Rudolph L, et al. (2008). Diagnosis and treatment of hepatocellular carcinoma. Gastroenterology 134:1752–63. 18471552 10.1053/j.gastro.2008.02.090

[CIT0029] Fang Y, Tan J, Lim S, et al. (2018). Rupturing cancer cells by the expansion of functionalized stimuli-responsive hydrogels. NPG Asia Mater 10:e465.

[CIT0032] Gajendiran M, Jo H, Kim K, et al. (2019). Green synthesis of multifunctional PEG-carboxylate π back-bonded gold nanoconjugates for breast cancer treatment. Int J Nanomedicine 14:819–34. 30774336 10.2147/IJN.S190946 PMC6354699

[CIT0033] Gao B, Luo J, Liu Y, et al. (2021). Intratumoral administration of thermosensitive hydrogel co-loaded with norcantharidin nanoparticles and doxorubicin for the treatment of hepatocellular carcinoma. Int J Nanomedicine 16:4073–85. 34163160 10.2147/IJN.S308057 PMC8214546

[CIT0034] Gao Z-G, Fain HD, Rapoport N. (2005). Controlled and targeted tumor chemotherapy by micellar-encapsulated drug and ultrasound. J Control Release 102:203–22. 15653146 10.1016/j.jconrel.2004.09.021

[CIT0035] Gharekhani H, Olad A, Mirmohseni A, et al. (2017). Superabsorbent hydrogel made of NaAlg-g-poly(AA-co-AAm) and rice husk ash: synthesis, characterization, and swelling kinetic studies. Carbohydr Polym 168:1–13. 28457428 10.1016/j.carbpol.2017.03.047

[CIT0036] GhavamiNejad A, SamariKhalaj M, Aguilar LE, et al. (2016). pH/NIR light-controlled multidrug release via a mussel-inspired nanocomposite hydrogel for chemo-photothermal cancer therapy. Sci Rep 6:1–12. 27646591 10.1038/srep33594 PMC5028867

[CIT0037] Godiya CB, Cheng X, Li D, et al. (2019). Carboxymethyl cellulose/polyacrylamide composite hydrogel for cascaded treatment/reuse of heavy metal ions in wastewater. J Hazard Mater 364:28–38. 30336333 10.1016/j.jhazmat.2018.09.076

[CIT0038] Hacker MC, Krieghoff J, Mikos AG. (2019). Synthetic polymers. In: Principles of regenerative medicine. Elsevier, 559–90.

[CIT0039] Hao Y, Lin CC. (2014). Degradable thiol-acrylate hydrogels as tunable matrices for three-dimensional hepatic culture. J Biomed Mater Res A 102:3813–27. 24288169 10.1002/jbm.a.35044

[CIT0040] Hu W, Wang Z, Xiao Y, et al. (2019). Advances in crosslinking strategies of biomedical hydrogels. Biomater Sci 7:843–55. 30648168 10.1039/c8bm01246f

[CIT0041] Huang X, Jain PK, El-Sayed IH, et al. (2008). Plasmonic photothermal therapy (PPTT) using gold nanoparticles. Lasers Med Sci 23:217–28. 17674122 10.1007/s10103-007-0470-x

[CIT0042] Hyun H, Park M, Jo G, et al. (2019). Photo-cured glycol chitosan hydrogel for ovarian cancer drug delivery. Mar Drugs 17:41. 30634553 10.3390/md17010041 PMC6356222

[CIT0043] Iizawa T, Taketa H, Maruta M, et al. (2007). Synthesis of porous poly (N‐isopropylacrylamide) gel beads by sedimentation polymerization and their morphology. J Appl Polym Sci 104:842–50.

[CIT0044] Jamal A, Shahzadi L, Ahtzaz S, et al. (2018). Identification of anti-cancer potential of doxazocin: loading into chitosan based biodegradable hydrogels for on-site delivery to treat cervical cancer. Mater Sci Eng C 82:102–9. 10.1016/j.msec.2017.08.054 29025638

[CIT0045] Jang SH, Wientjes MG, Lu D, et al. (2003). Drug delivery and transport to solid tumors. Pharm Res 20:1337–50. 14567626 10.1023/a:1025785505977

[CIT0046] Jeong H-S, Hwang H, Oh P-S, et al. (2016). Effect of high-intensity focused ultrasound on drug release from doxorubicin-loaded PEGylated liposomes and therapeutic effect in colorectal cancer murine models. Ultrasound Med Biol 42:947–55. 26795498 10.1016/j.ultrasmedbio.2015.12.005

[CIT0047] Jin RM, Yang J, Zhao DH, et al. (2019). Hollow gold nanoshells-incorporated injectable genetically engineered hydrogel for sustained chemo-photothermal therapy of tumor. J Nanobiotechnol 17:1–16. 10.1186/s12951-019-0532-9 PMC6749695 31530285

[CIT0048] Karimi AR, Tarighatjoo M, Nikravesh G. (2017). 1,3,5-Triazine-2,4,6-tribenzaldehyde derivative as a new crosslinking agent for synthesis of pH-thermo dual responsive chitosan hydrogels and their nanocomposites: swelling properties and drug release behavior. Int J Biol Macromol 105:1088–95. 28739410 10.1016/j.ijbiomac.2017.07.128

[CIT0049] Kasiński A, Zielińska-Pisklak M, Oledzka E, et al. (2020). Smart hydrogels – synthetic stimuli-responsive antitumor drug release systems. Int J Nanomedicine 15:4541–72. 32617004 10.2147/IJN.S248987 PMC7326401

[CIT0050] Khan S, Akhtar N, Minhas MU, et al. (2019). pH/thermo-dual responsive tunable in situ cross-linkable depot injectable hydrogels based on poly (N-isopropylacrylamide)/carboxymethyl chitosan with potential of controlled localized and systemic drug delivery. AAPS PharmSciTech 20:1–16. 10.1208/s12249-019-1328-9 30790143

[CIT0051] Kumar A, Han SS. (2017). PVA-based hydrogels for tissue engineering: a review. Int J Polym Mater Polym Biomater 66:159–82.

[CIT0052] Lee DS, Kim BS, Huynh CT. (2013). Drug delivery system for treatment of liver cancer based on interventional injection of temperature and pH-sensitive hydrogel. Google Patents.

[CIT0053] Lee DS, Kim BS, Nguyen MK. (2013). Poly (amidoamine) oligomer hydrogel for drug delivery and drug carrier using the same. Google Patents.

[CIT0054] Lee SC, Kwon IK, Park K. (2013). Hydrogels for delivery of bioactive agents: a historical perspective. Adv Drug Deliv Rev 65:17–20. 22906864 10.1016/j.addr.2012.07.015 PMC3514585

[CIT0055] Li F, Yang H, Bie N, et al. (2017). Zwitterionic temperature/redox-sensitive nanogels for near-infrared light-triggered synergistic thermo-chemotherapy. ACS Appl Mater Interfaces 9:23564–73. 28665111 10.1021/acsami.7b08047

[CIT0056] Li J, Mooney DJ. (2016). Designing hydrogels for controlled drug delivery. Nat Rev Mater 1:1–17. 10.1038/natrevmats.2016.71 PMC5898614 29657852

[CIT0057] Li L, Cao L, Xiang X, et al. (2022). ROS‐catalytic transition‐metal‐based enzymatic nanoagents for tumor and bacterial eradication. Adv Funct Mater 32:2107530.

[CIT0058] Lim HL, Hwang Y, Kar M, et al. (2014). Smart hydrogels as functional biomimetic systems. Biomater Sci 2:603–18. 32481841 10.1039/c3bm60288e

[CIT0059] Liu H, Rong L, Wang B, et al. (2017). Facile fabrication of redox/pH dual stimuli responsive cellulose hydrogel. Carbohydr Polym 176:299–306. 28927612 10.1016/j.carbpol.2017.08.085

[CIT0060] Liu XJ, Ren XY, Guan S, et al. (2015). Highly stretchable and tough double network hydrogels via molecular stent. Eur Polym J 73:149–61.

[CIT0061] Lu J, Wang J, Ling D. (2018). Surface engineering of nanoparticles for targeted delivery to hepatocellular carcinoma. Small 14:1702037. 10.1002/smll.201702037 29251419

[CIT0062] Lv Q, He C, Quan F, et al. (2018). DOX/IL-2/IFN-γ co-loaded thermo-sensitive polypeptide hydrogel for efficient melanoma treatment. Bioact Mater 3:118–28. 29744449 10.1016/j.bioactmat.2017.08.003 PMC5935762

[CIT0063] Makhmalzadeh BS, Molavi O, Vakili MR, et al. (2018). Functionalized caprolactone-polyethylene glycol based thermo-responsive hydrogels of silibinin for the treatment of malignant melanoma. J Pharm Pharm Sci 21:143–59. 29789104 10.18433/jpps29726

[CIT0064] Manga RD, Jha PK. (2017). Mathematical models for controlled drug release through pH-responsive polymeric hydrogels. J Pharm Sci 106:629–38. 27890245 10.1016/j.xphs.2016.10.019

[CIT0065] Manouras T, Vamvakaki M. (2017). Field responsive materials: photo-, electro-, magnetic-and ultrasound-sensitive polymers. Polym Chem 8:74–96.

[CIT0066] Maolin Z, Jun L, Min Y, et al. (2000). The swelling behavior of radiation prepared semi-interpenetrating polymer networks composed of polyNIPAAm and hydrophilic polymers. Radiat Phys Chem 58:397–400.

[CIT0067] Martin N, Youssef G. (2018). Dynamic properties of hydrogels and fiber-reinforced hydrogels. J Mech Behav Biomed Mater 85:194–200. 29908487 10.1016/j.jmbbm.2018.06.008

[CIT0068] Meyer DE, Chilkoti A. (1999). Purification of recombinant proteins by fusion with thermally-responsive polypeptides. Nat Biotechnol 17:1112–5. 10545920 10.1038/15100

[CIT0069] Miller KD, Nogueira L, Mariotto AB, et al. (2019). Cancer treatment and survivorship statistics, 2019. CA Cancer J Clin 69:363–85. 31184787 10.3322/caac.21565

[CIT0070] Minhas MU, Ahmad M, Anwar J, et al. (2018). Synthesis and characterization of biodegradable hydrogels for oral delivery of 5‐fluorouracil targeted to colon: screening with preliminary in vivo studies. Adv Polym Technol 37:221–9.

[CIT0071] Mittal S, El-Serag HB, Sada YH, et al. (2016). Hepatocellular carcinoma in the absence of cirrhosis in United States veterans is associated with nonalcoholic fatty liver disease. Clin Gastroenterol Hepatol 14:124–31. 26196445 10.1016/j.cgh.2015.07.019 PMC4690789

[CIT0072] Miyata T, Uragami T, Nakamae K. (2002). Biomolecule-sensitive hydrogels. Adv Drug Deliv Rev 54:79–98. 11755707 10.1016/s0169-409x(01)00241-1

[CIT0073] Na K, Park KH, Kim SW, et al. (2000). Self-assembled hydrogel nanoparticles from curdlan derivatives: characterization, anti-cancer drug release and interaction with a hepatoma cell line (HepG2). J Control Release 69:225–36. 11064130 10.1016/s0168-3659(00)00256-x

[CIT0074] Nagakawa Y, Yi S‐Q, Takishita C, et al. (2020). Precise anatomical resection based on structures of nerve and fibrous tissue around the superior mesenteric artery for mesopancreas dissection in pancreaticoduodenectomy for pancreatic cancer. J Hepatobiliary Pancreat Sci 27:342–51. 32048456 10.1002/jhbp.725

[CIT0075] Nakamura YA, Okuyama S, Furusawa A, et al. (2019). Near-infrared photoimmunotherapy through bone. Cancer Sci 110:3689–94. 31553485 10.1111/cas.14203 PMC6890452

[CIT0076] Nandwana V, Ryoo S-R, Zheng T, et al. (2019). Magnetic nanostructure-coated thermoresponsive hydrogel nanoconstruct as a smart multimodal theranostic platform. ACS Biomater Sci Eng 5:3049–59. 33405658 10.1021/acsbiomaterials.9b00361

[CIT0077] Norouzi M, Nazari B, Miller DW. (2016). Injectable hydrogel-based drug delivery systems for local cancer therapy. Drug Discov Today 21:1835–49. 27423369 10.1016/j.drudis.2016.07.006

[CIT0078] Ojugo ASE, McSheehy PMJ, McIntyre DJO, et al. (1999). Measurement of the extracellular pH of solid tumours in mice by magnetic resonance spectroscopy: a comparison of exogenous 19F and 31P probes. NMR Biomed 12:495–504. 10668042 10.1002/(sici)1099-1492(199912)12:8<495::aid-nbm594>3.0.co;2-k

[CIT0079] Pan A, Wang Z, Chen B, et al. (2018). Localized co-delivery of collagenase and trastuzumab by thermosensitive hydrogels for enhanced antitumor efficacy in human breast xenograft. Drug Deliv 25:1495–503. 29943651 10.1080/10717544.2018.1474971 PMC6058501

[CIT0080] Peng C-L, Shih Y-H, Liang K-S, et al. (2013). Development of in situ forming thermosensitive hydrogel for radiotherapy combined with chemotherapy in a mouse model of hepatocellular carcinoma. Mol Pharm 10:1854–64. 23485019 10.1021/mp3006424

[CIT0081] Peng M, Xu S, Zhang Y, et al. (2014). Thermosensitive injectable hydrogel enhances the antitumor effect of embelin in mouse hepatocellular carcinoma. J Pharm Sci 103:965–73. 24481745 10.1002/jps.23885

[CIT0082] Pennisi G, Celsa C, Giammanco A, et al. (2019). The burden of hepatocellular carcinoma in non-alcoholic fatty liver disease: screening issue and future perspectives. Int J Mol Sci 20:5613. 31717576 10.3390/ijms20225613 PMC6887792

[CIT0083] Pereira RF, Bártolo PJ. (2014). Photopolymerizable hydrogels in regenerative medicine and drug delivery. Future Medicine. London, UK.

[CIT0084] Persi E, Duran-Frigola M, Damaghi M, et al. (2018). Systems analysis of intracellular pH vulnerabilities for cancer therapy. Nat Commun 9:1–11. 30065243 10.1038/s41467-018-05261-x PMC6068141

[CIT0085] Puente Pdl, Fettig N, Luderer MJ, et al. (2018). Injectable hydrogels for localized chemotherapy and radiotherapy in brain tumors. J Pharm Sci 107:922–33. 29162424 10.1016/j.xphs.2017.10.042 PMC6093750

[CIT0086] Qian K-Y, Song Y, Yan X, et al. (2020). Injectable ferrimagnetic silk fibroin hydrogel for magnetic hyperthermia ablation of deep tumor. Biomaterials 259:120299. 32827797 10.1016/j.biomaterials.2020.120299

[CIT0087] Qin S-Y, Zhang A-Q, Cheng S-X, et al. (2017). Drug self-delivery systems for cancer therapy. Biomaterials 112:234–47. 27768976 10.1016/j.biomaterials.2016.10.016

[CIT0088] Qiu Y, Park K. (2001). Environment-sensitive hydrogels for drug delivery. Adv Drug Deliv Rev 53:321–39. 11744175 10.1016/s0169-409x(01)00203-4

[CIT0089] Qu J, Zhao X, Liang Y, et al. (2018). Antibacterial adhesive injectable hydrogels with rapid self-healing, extensibility and compressibility as wound dressing for joints skin wound healing. Biomaterials 183:185–99. 30172244 10.1016/j.biomaterials.2018.08.044

[CIT0090] Qu J, Zhao X, Liang Y, et al. (2019). Degradable conductive injectable hydrogels as novel antibacterial, anti-oxidant wound dressings for wound healing. Chem Eng J 362:548–60.

[CIT0091] Qu J, Zhao X, Ma PX, et al. (2017). pH-responsive self-healing injectable hydrogel based on N-carboxyethyl chitosan for hepatocellular carcinoma therapy. Acta Biomater 58:168–80. 28583902 10.1016/j.actbio.2017.06.001

[CIT0092] Qu J, Zhao X, Ma PX, et al. (2018). Injectable antibacterial conductive hydrogels with dual response to an electric field and pH for localized “smart” drug release. Acta Biomater 72:55–69. 29555459 10.1016/j.actbio.2018.03.018

[CIT0093] Raza F, Zhu Y, Chen L, et al. (2019). Paclitaxel-loaded pH responsive hydrogel based on self-assembled peptides for tumor targeting. Biomater Sci 7:2023–36. 30839983 10.1039/c9bm00139e

[CIT0094] Ren Y, Li X, Han B, et al. (2019). Improved anti-colorectal carcinomatosis effect of tannic acid co-loaded with oxaliplatin in nanoparticles encapsulated in thermosensitive hydrogel. Eur J Pharm Sci 128:279–89. 30553061 10.1016/j.ejps.2018.12.007

[CIT0095] Ringelhan M, Pfister D, O'Connor T, et al. (2018). The immunology of hepatocellular carcinoma. Nat Immunol 19:222–32. 29379119 10.1038/s41590-018-0044-z

[CIT0096] Rosenblum D, Joshi N, Tao W, et al. (2018). Progress and challenges towards targeted delivery of cancer therapeutics. Nat Commun 9:1–12. 29650952 10.1038/s41467-018-03705-y PMC5897557

[CIT0097] Rosenthal A, Barry JJ, Sahatjian R. (2006). Triggered release hydrogel drug delivery system. Google Patents.

[CIT0098] Rosiak J, Rucinska-Rybus A, Pekala W. (1989). Method of manufacturing hydrogel dressings. Google Patents.

[CIT0099] Ruan C, Liu C, Hu H, et al. (2019). NIR-II light-modulated thermosensitive hydrogel for light-triggered cisplatin release and repeatable chemo-photothermal therapy. Chem Sci 10:4699–706. 31123581 10.1039/c9sc00375d PMC6496981

[CIT0100] Russo E, Villa C. (2019). Poloxamer hydrogels for biomedical applications. Pharmaceutics 11:671. 31835628 10.3390/pharmaceutics11120671 PMC6955690

[CIT0101] Sahraei R, Ghaemy M. (2017). Synthesis of modified gum tragacanth/graphene oxide composite hydrogel for heavy metal ions removal and preparation of silver nanocomposite for antibacterial activity. Carbohydr Polym 157:823–33. 27987996 10.1016/j.carbpol.2016.10.059

[CIT0102] Salimi F, Dilmaghani KA, Alizadeh E, et al. (2018). Enhancing cisplatin delivery to hepatocellular carcinoma HepG2 cells using dual sensitive smart nanocomposite. Artif Cells Nanomed Biotechnol 46:949–58. 28687054 10.1080/21691401.2017.1349777

[CIT0103] Scharovsky OG, Mainetti LE, Rozados VR. (2009). Metronomic chemotherapy: changing the paradigm that more is better. Curr Oncol 16:7–15. 10.3747/co.v16i2.420 PMC2669231 19370174

[CIT0104] Schiller JL, Lai SK. (2020). Tuning barrier properties of biological hydrogels. ACS Appl Bio Mater 3:2875–90. 10.1021/acsabm.0c00187 35025336

[CIT0105] Senft D, Qi J, Ze'ev AR. (2018). Ubiquitin ligases in oncogenic transformation and cancer therapy. Nat Rev Cancer 18:69–88. 29242641 10.1038/nrc.2017.105 PMC6054770

[CIT0106] Sepantafar M, Maheronnaghsh R, Mohammadi H, et al. (2017). Engineered hydrogels in cancer therapy and diagnosis. Trends Biotechnol 35:1074–87. 28734545 10.1016/j.tibtech.2017.06.015

[CIT0107] Sharma R. (2020). Descriptive epidemiology of incidence and mortality of primary liver cancer in 185 countries: evidence from GLOBOCAN 2018. Jpn J Clin Oncol 50:1370–9. 32719873 10.1093/jjco/hyaa130

[CIT0108] Shen W, Chen X, Luan J, et al. (2017). Sustained codelivery of cisplatin and paclitaxel via an injectable prodrug hydrogel for ovarian cancer treatment. ACS Appl Mater Interfaces 9:40031–46. 29131563 10.1021/acsami.7b11998

[CIT0109] Shih T‐Y, Blacklow SO, Li AW, et al. (2018). Injectable, tough alginate cryogels as cancer vaccines. Adv Healthcare Mater 7:1701469. 10.1002/adhm.201701469 PMC6467206 29441705

[CIT0110] Sinha V, Chakma S. (2019). Advances in the preparation of hydrogel for wastewater treatment: a concise review. J Environ Chem Eng 7:103295.

[CIT0111] Siqueira NM, Cirne MFR, Immich MF, Poletto F. (2018). Stimuli-responsive polymeric hydrogels and nanogels for drug delivery applications. In: Stimuli responsive polymeric nanocarriers for drug delivery applications. Vol. 1. Elsevier, 343–74.

[CIT0112] Smith TT, Moffett HF, Stephan SB, et al. (2017). Biopolymers codelivering engineered T cells and STING agonists can eliminate heterogeneous tumors. J Clin Invest 127:2176–91. 28436934 10.1172/JCI87624 PMC5451231

[CIT0113] Society AC. (2018). Polyphosphazenes in biomedicine, engineering, and pioneering synthesis. Washington, DC: ACS Publications.

[CIT0114] Song SS, Kim HH, Yil WY. (1996). Biodegradable hydrogel copolymer as drug delivery matrix. Google Patents.

[CIT0115] Starley BQ, Calcagno CJ, Harrison SA. (2010). Nonalcoholic fatty liver disease and hepatocellular carcinoma: a weighty connection. Hepatology 51:1820–32. 20432259 10.1002/hep.23594

[CIT0116] Sultana T, Van Hai H, Abueva C, et al. (2019). TEMPO oxidized nano-cellulose containing thermo-responsive injectable hydrogel for post-surgical peritoneal tissue adhesion prevention. Mater Sci Eng C Mater Biol Appl 102:12–21. 31146982 10.1016/j.msec.2019.03.110

[CIT0117] Surrao DC, Arasu Y, Ekberg JAK, et al. (2020). Blended, crosslinked alginate-methylcellulose hydrogels for encapsulation and delivery of olfactory ensheathing cells. Materialia 10:100654.

[CIT0118] Takimoto CH, Awada A. (2008). Safety and anti-tumor activity of sorafenib (Nexavar) in combination with other anti-cancer agents: a review of clinical trials. Cancer Chemother Pharmacol 61:535–48. 18026728 10.1007/s00280-007-0639-9

[CIT0119] Tavakoli S, Klar AS. (2020). Advanced hydrogels as wound dressings. Biomolecules 10:1169. 32796593 10.3390/biom10081169 PMC7464761

[CIT0120] Tsirigotis-Maniecka M, Szyk-Warszyńska L, Maniecki Ł, et al. (2021). Tailoring the composition of hydrogel particles for the controlled delivery of phytopharmaceuticals. Eur Polym J 151:110429.

[CIT0121] Ullah F, Othman MBH, Javed F, et al. (2015). Classification, processing and application of hydrogels: a review. Mater Sci Eng C 57:414–33. 10.1016/j.msec.2015.07.053 26354282

[CIT0122] Varshosaz J, Farzan M. (2015). Nanoparticles for targeted delivery of therapeutics and small interfering RNAs in hepatocellular carcinoma. World J Gastroenterol 21:12022–41. 26576089 10.3748/wjg.v21.i42.12022 PMC4641122

[CIT0123] Velde BL, Westhuyzen J, Awad N, et al. (2019). Late toxicities of prostate cancer radiotherapy with and without hydrogel SpaceAOR insertion. J Med Imaging Radiat Oncol 63:836–41. 31520465 10.1111/1754-9485.12945

[CIT0124] Vermonden T, Klumperman B. (2015). The past, present and future of hydrogels. European Polymer Journal 72:341–3.

[CIT0125] Vermonden T, Censi R, Hennink WE. (2012). Hydrogels for protein delivery. Chem Rev 112:2853–88. 22360637 10.1021/cr200157d

[CIT0126] Wan J, Geng S, Zhao H, et al. (2016). Doxorubicin-induced co-assembling nanomedicines with temperature-sensitive acidic polymer and their in-situ-forming hydrogels for intratumoral administration. J Control Release 235:328–36. 27282415 10.1016/j.jconrel.2016.06.009

[CIT0127] Wang C, Willner B, Willner I. (2021). Redox-responsive and light-responsive DNA-based hydrogels and their applications. React Funct Polym 166:104983.

[CIT0128] Wang H, Liu X, Wang Y, et al. (2015). Doxorubicin conjugated phospholipid prodrugs as smart nanomedicine platforms for cancer therapy. J Mater Chem B 3:3297–305. 32262324 10.1039/c4tb01984a

[CIT0129] Wang X, Qiao L, Yu X, et al. (2019). Controllable formation of ternary inorganic-supramolecular-polymeric hydrogels by amidation-fueled self-assembly and enzymatic post-cross-linking for ultrasound theranostic. ACS Biomater Sci Eng 5:5888–96. 33405679 10.1021/acsbiomaterials.9b01065

[CIT0130] Wang Y, Han B, Shi R, et al. (2013). Preparation and characterization of a novel hybrid hydrogel shell for localized photodynamic therapy. J Mater Chem B 1:6411–7. 32261339 10.1039/c3tb20779j

[CIT0131] Wang Y. (2018). Programmable hydrogels. Biomaterials 178:663–80. 29549970 10.1016/j.biomaterials.2018.03.008 PMC6054804

[CIT0132] Wei C, Dong X, Zhang Y, et al. (2018). Simultaneous fluorescence imaging monitoring of the programmed release of dual drugs from a hydrogel-carbon nanotube delivery system. Sens Actuators B 273:264–75.

[CIT0133] Wei W, Li H, Yin C, et al. (2020). Research progress in the application of in situ hydrogel system in tumor treatment. Drug Deliv 27:460–8. 32166987 10.1080/10717544.2020.1739171 PMC7144265

[CIT0134] Wu J, Jiang W, Tian R, et al. (2016). Facile synthesis of magnetic-/pH-responsive hydrogel beads based on Fe_3_O_4_ nanoparticles and chitosan hydrogel as MTX carriers for controlled drug release. J Biomater Sci Polym Ed 27:1553–68. 27464586 10.1080/09205063.2016.1218212

[CIT0135] Wu Q, He Z, Wang X, et al. (2019). Cascade enzymes within self-assembled hybrid nanogel mimicked neutrophil lysosomes for singlet oxygen elevated cancer therapy. Nat Commun 10:1–14. 30651559 10.1038/s41467-018-08234-2 PMC6335431

[CIT0136] Wu R-S, Lin J, Xing Y-M, et al. (2019). pH-sensitive black phosphorous-incorporated hydrogel as novel implant for cancer treatment. J Pharm Sci 108:2542–51. 30876860 10.1016/j.xphs.2019.03.003

[CIT0137] Xu C, Yan Y, Tan J, et al. (2019). Biodegradable nanoparticles of polyacrylic acid-stabilized amorphous CaCO_3_ for tunable pH‐responsive drug delivery and enhanced tumor inhibition. Adv Funct Mater 29:1808146.

[CIT0138] Xu X, Huang Z, Huang Z, et al. (2017). Injectable, NIR/pH-responsive nanocomposite hydrogel as long-acting implant for chemophotothermal synergistic cancer therapy. ACS Appl Mater Interfaces 9:20361–75. 28532154 10.1021/acsami.7b02307

[CIT0139] Yallapu MM, Jaggi M, Chauhan SC. (2011). Design and engineering of nanogels for cancer treatment. Drug Discov Today 16:457–63. 21414419 10.1016/j.drudis.2011.03.004 PMC3095726

[CIT0140] Yamaguchi N, Zhang L, Chae B-S, et al. (2007). Growth factor mediated assembly of cell receptor-responsive hydrogels. J Am Chem Soc 129:3040–1. 17315874 10.1021/ja0680358 PMC2606044

[CIT0141] Yan J-J, Liao J-Z, Lin J-S, et al. (2015). Active radar guides missile to its target: receptor-based targeted treatment of hepatocellular carcinoma by nanoparticulate systems. Tumour Biol 36:55–67. 25424700 10.1007/s13277-014-2855-3

[CIT0142] Yan X, Sun T, Song Y, et al. (2022). In situ thermal-responsive magnetic hydrogel for multidisciplinary therapy of hepatocellular carcinoma. Nano Lett 22:2251–60. 35254836 10.1021/acs.nanolett.1c04413

[CIT0143] Yang L, Chu JS, Fix JA. (2002). Colon-specific drug delivery: new approaches and in vitro/in vivo evaluation. Int J Pharm 235:1–15. 11879735 10.1016/s0378-5173(02)00004-2

[CIT0144] Yoshida R, Uchida K, Kaneko Y, et al. (1995). Comb-type grafted hydrogels with rapid deswelling response to temperature changes. Nature 374:240–2.

[CIT0145] Yu S, Wang C, Yu J, et al. (2018). Injectable bioresponsive gel depot for enhanced immune checkpoint blockade. Adv Mater 30:1801527. 10.1002/adma.201801527 29786888

[CIT0146] Yu S, Wei S, Liu L, et al. (2019). Enhanced local cancer therapy using a CA4P and CDDP co-loaded polypeptide gel depot. Biomater Sci 7:860–6. 30698593 10.1039/c8bm01442f

[CIT0147] Zhang F, Gong S, Wu J, et al. (2017). CXCR4-targeted and redox responsive dextrin nanogel for metastatic breast cancer therapy. Biomacromolecules 18:1793–802. 28445650 10.1021/acs.biomac.7b00208

[CIT0148] Zhang J, Preda DV, Vasquez KO, et al. (2012). A fluorogenic near-infrared imaging agent for quantifying plasma and local tissue renin activity in vivo and ex vivo. Am J Physiol Renal Physiol 303:F593–F603. 22674025 10.1152/ajprenal.00361.2011 PMC3423114

[CIT0149] Zhang J, Yang L, Huang F, et al. (2021). Multifunctional hybrid hydrogel enhanced antitumor therapy through multiple destroying DNA functions by a triple‐combination synergistic therapy. Adv Healthcare Mater 10:2101190. 10.1002/adhm.202101190 34382378

[CIT0150] Zhang L, Zeng Y, Cheng Z. (2016). Removal of heavy metal ions using chitosan and modified chitosan: a review. J Mol Liq 214:175–91.

[CIT0151] Zhang N, Xu X, Zhang X, et al. (2016). Nanocomposite hydrogel incorporating gold nanorods and paclitaxel-loaded chitosan micelles for combination photothermal-chemotherapy. Int J Pharm 497:210–21. 26608619 10.1016/j.ijpharm.2015.11.032

[CIT0152] Zhao D, Song H, Zhou X, et al. (2019). Novel facile thermosensitive hydrogel as sustained and controllable gene release vehicle for breast cancer treatment. Eur J Pharm Sci 134:145–52. 30926401 10.1016/j.ejps.2019.03.021

[CIT0153] Zhao M, Bozzato E, Joudiou N, et al. (2019). Codelivery of paclitaxel and temozolomide through a photopolymerizable hydrogel prevents glioblastoma recurrence after surgical resection. J Control Release 309:72–81. 31306678 10.1016/j.jconrel.2019.07.015

[CIT0154] Zhao W, Jin X, Cong Y, et al. (2013). Degradable natural polymer hydrogels for articular cartilage tissue engineering. J Chem Technol Biotechnol 88:327–39.

[CIT0155] Zhao X, Li X, Zhang P, et al. (2018). Tip-loaded fast-dissolving microneedle patches for photodynamic therapy of subcutaneous tumor. J Control Release 286:201–9. 30056119 10.1016/j.jconrel.2018.07.038

[CIT0156] Zheng Y, Wang W, Zhao J, et al. (2019). Preparation of injectable temperature-sensitive chitosan-based hydrogel for combined hyperthermia and chemotherapy of colon cancer. Carbohydr Polym 222:115039. 31320053 10.1016/j.carbpol.2019.115039

[CIT0157] Zhou J, Yu G, Huang F. (2017). Supramolecular chemotherapy based on host–guest molecular recognition: a novel strategy in the battle against cancer with a bright future. Chem Soc Rev 46:7021–53. 28980674 10.1039/c6cs00898d

[CIT0158] Zhu L, Li M, Liu X, et al. (2017). Drug-loaded PLGA electrospraying porous microspheres for the local therapy of primary lung cancer via pulmonary delivery. ACS Omega 2:2273–9. 30023660 10.1021/acsomega.7b00456 PMC6044788

